# Embryo-scale reverse genetics at single-cell resolution

**DOI:** 10.1038/s41586-023-06720-2

**Published:** 2023-11-15

**Authors:** Lauren M. Saunders, Sanjay R. Srivatsan, Madeleine Duran, Michael W. Dorrity, Brent Ewing, Tor H. Linbo, Jay Shendure, David W. Raible, Cecilia B. Moens, David Kimelman, Cole Trapnell

**Affiliations:** 1 Department of Genome Sciences, University of Washington, Seattle, WA USA; 2 Department of Biological Structure, University of Washington, Seattle, WA USA; 3 Brotman Baty Institute for Precision Medicine, University of Washington, Seattle, WA USA; 4 Howard Hughes Medical Institute, Seattle, WA USA; 5 Allen Discovery Center for Cell Lineage Tracing, Seattle, WA USA; 6 Fred Hutchinson Cancer Center, Seattle, WA USA; 7 Department of Biochemistry, University of Washington, Seattle, WA USA

**Keywords:** Embryogenesis, Transcriptomics, Zebrafish, Development

## Abstract

The maturation of single-cell transcriptomic technologies has facilitated the generation of comprehensive cellular atlases from whole embryos^1–4^. A majority of these data, however, has been collected from wild-type embryos without an appreciation for the latent variation that is present in development. Here we present the ‘zebrafish single-cell atlas of perturbed embryos’: single-cell transcriptomic data from 1,812 individually resolved developing zebrafish embryos, encompassing 19 timepoints, 23 genetic perturbations and a total of 3.2 million cells. The high degree of replication in our study (eight or more embryos per condition) enables us to estimate the variance in cell type abundance organism-wide and to detect perturbation-dependent deviance in cell type composition relative to wild-type embryos. Our approach is sensitive to rare cell types, resolving developmental trajectories and genetic dependencies in the cranial ganglia neurons, a cell population that comprises less than 1% of the embryo. Additionally, time-series profiling of individual mutants identified a group of *brachyury*-independent cells with strikingly similar transcriptomes to notochord sheath cells, leading to new hypotheses about early origins of the skull. We anticipate that standardized collection of high-resolution, organism-scale single-cell data from large numbers of individual embryos will enable mapping of the genetic dependencies of zebrafish cell types, while also addressing longstanding challenges in developmental genetics, including the cellular and transcriptional plasticity underlying phenotypic diversity across individuals.

## Abstract (web summary)

We present the ‘zebrafish single-cell atlas of perturbed embryos’, single-cell trancriptomic data of developing zebrafish embryos across various timepoints and with genetic perturbations.

## Main

Understanding how each gene in our genome contributes to our individual phenotypes during embryogenesis is a fundamental goal of developmental genetics. Genetic screens in multicellular animals have enabled the dissection of diverse developmental processes, illuminating the functions of thousands of genes. Although advances in automation, imaging and genetic tools have increased the sophistication of phenotyping and yielded new insights into vertebrate development, phenotyping remains a substantial bottleneck in characterizing gene function. Single-cell RNA sequencing (scRNA-seq) applied at whole-embryo scale offers a comprehensive means of simultaneously measuring molecular and cellular phenotypes^1–4^. However, realizing this promise requires overcoming several challenges: sequencing a very large number of cells through developmental time, rapidly generating mutant embryos and sampling many individuals to account for biological variability during embryogenesis. These challenges have, until now, limited analyses to few genetic perturbations in comparatively less complex animals or at early stages of development.

Recent technological advances have created an opportunity to overcome these challenges, spurring a new era of developmental genomics. Combinatorial cellular indexing, or ‘sci-seq’, profiles the transcriptomes of millions of nuclei in one experiment, enabling embryo-scale analyses^2^. Labelling techniques that ‘hash’ cells or nuclei from distinct samples allow one to multiplex specimens or whole embryos together^5^, facilitating the analysis of many individuals. Parallel advances in CRISPR–Cas9 mutagenesis now enable programmatic, highly efficient genome editing at the F0 stage^6^, circumventing the generation time required to create mutant embryos.

Here, we describe the application of these three technologies to zebrafish, a model organism that develops rapidly, exhibits extensive cell type diversity and is made up of a relatively small number of cells. The ‘zebrafish single-cell atlas of perturbed embryos’ (ZSCAPE) constitutes two major efforts: (1) the establishment of an annotated, individually resolved reference atlas, comprising 1,167 individuals and 1.2 million cells over 19 timepoints, filling a major gap in existing zebrafish atlases; and (2) the collection of perturbation data from 23 genetic perturbations over multiple timepoints, totalling 645 individuals and 2 million cells. By collecting many replicate embryos (eight or more embryos per condition), we implement statistical tests to systematically assess the gains and loss of cell types consequent to perturbation throughout the developing zebrafish. By comparing our harmonized reference and perturbation datasets, we dissect the genetic dependencies of rare cell types such as the the sensory neurons of the cranial ganglia, which comprise less than 1% of the cells in the organism. Finally, we leverage time-resolved, differential cell type abundance analysis to characterize a cryptic population of cranial cartilage, explicating new hypotheses regarding the evolutionary origins of the vertebrate skull. Together, our scalable approach is flexible, comprehensive, cost-effective and more uniform than conventional phenotyping strategies. We anticipate that this new experimental and analytical workflow will enable rapid, high-resolution phenotyping of whole animals to better understand the genetic dependencies of cell types in a developing organism.

## An atlas of individual embryos

**Extended Data Fig. 1:**
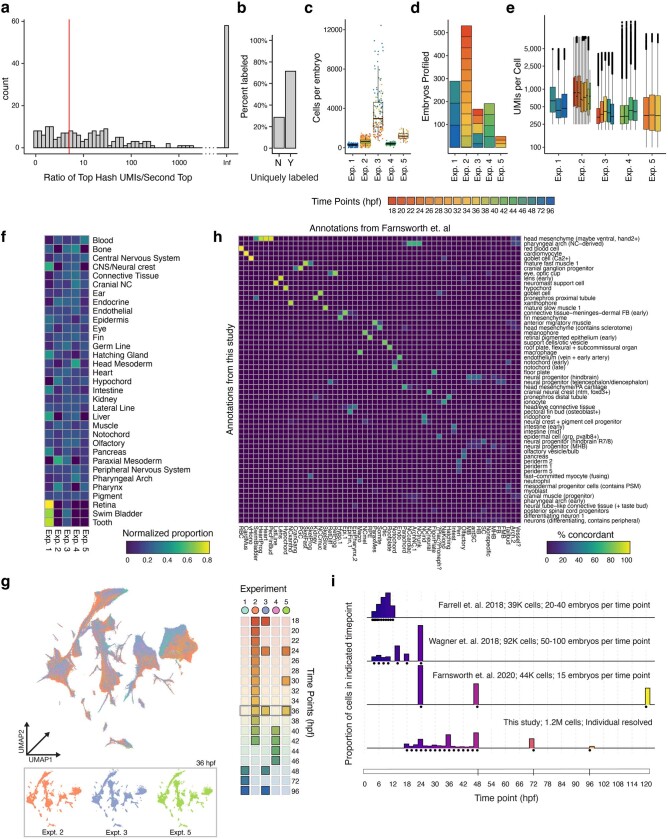
Experimental and QC metrics for the reference sci-RNA-seq data. **a**, Enrichment ratio distribution – the ratio between the counts for a cell’s top hash oligo and the second most abundant hash oligo after subtracting background hash molecules. Cells displaying a 5 fold enrichment (red line) of a single hash oligo were deemed uniquely labeled. **b**, Percentage of uniquely labeled cells (Y – uniquely labeled, N – not uniquely labeled). **c**-**e**, Reference dataset summary statistics displaying the number of (**c**) cells per embryo, (**d**) embryos and (**e**) UMIs recovered from each experiment. Plots are colored by the timepoint of embryo collection and timepoints are displayed as hours post-fertilization (hpf). **f**, Heatmap depicting the tissues to which cells from each experiment map. The count matrix was row and column normalized before visualization. **g**, UMAP embedding in 3-dimensions of the wildtype reference dataset colored by experiment of origin and plotting order randomized. Heatmap (right) shows which timepoints were contained within each experiment. Inset (below) displays 36 hpf timepoint, faceted by experiment. **h**, Heatmap depicting the percentage of each cell type in the Farnsworth^9^ dataset with nearest neighbors in this study at 24 hpf. Columns are annotations from Farnsworth et al. (2018), rows are annotations from this study and each column sums to 100%. Transcriptomes from the two datasets were restricted to a shared set of genes, and downsampled before alignment with Seurat. **i**, Cell-count mean/variance relationships for all cell types per individual embryo, collapsed by timepoint and ranked by means. Mean–variance relationships are computed via beta-binomial modeling of the variance, followed by significance testing on the variance observed over the variance expected based on mean cell abundance. Colors denote different cell types.

To robustly detect perturbation-dependent changes in cellular composition, we adapted sci-Plex^5^, a workflow for multiplexing thousands of samples during scRNA-seq, to barcode individual embryos and to capture single-nucleus transcriptomes from whole organisms (Methods). We optimized whole-embryo dissociations followed by oligonucleotide hashing to label each nucleus with an embryo-specific barcode, finding that we can unambiguously recover the embryo of origin for around 70% of cells passing quality control thresholds (Extended Data Fig. 1a,b and Supplementary Table 1).

Existing single-cell atlases of zebrafish development document the emergence of diverse cell types from 3.3 h (pregastrulation) to 5 days (late organogenesis) post-fertilization, in addition to a few selected mutants at a single timepoint^7–9^. While these datasets resolved diverse cellular states during zebrafish embryogenesis, each timepoint was a pool of embryos, thus masking heterogeneity between individuals. To assess variation resulting from gene knockouts, estimating the baseline heterogeneity present between individual wild-type embryos is critical. Moreover, after late segmentation (18 h post-fertilization (hpf)), intervals between sampling timepoints in these datasets were very sparse and therefore were not well resolved for key differentiation events during organogenesis. Thus, we first set out to establish a more high-resolution reference atlas with individual embryo resolution and fine-grained timepoint sampling.

**Fig. 1:**
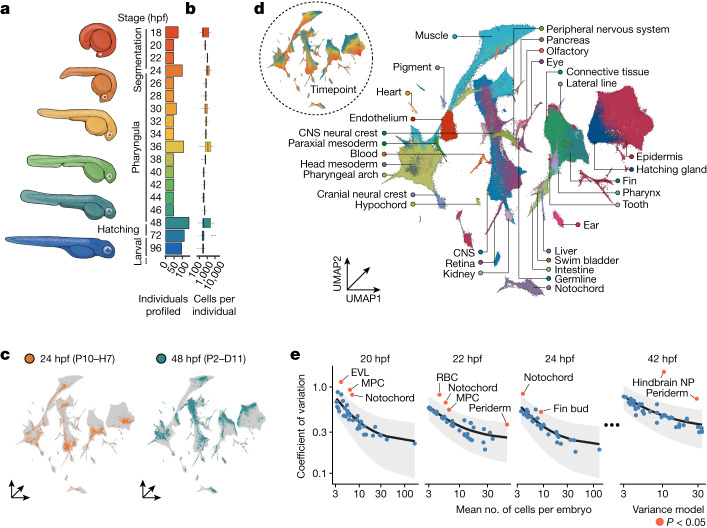
Collection of an individual-resolved single-cell zebrafish atlas using oligonucleotide hashing. **a**,**b**, Number of individuals (**a**, right) and cells per individual embryo (**b**) profiled from each developmental timepoint. Thick horizontal lines show medians, box edges delineate first and third quartiles, whiskers extend to ±1.5× interquartile range and dots show outliers. Representative drawings for select stages are shown (left) with colours matching timepoints in the bar graph. **c**, Cells originating from two individual embryos from 24 hpf (left) and 48 hpf (right) titled with the hash oligonucleotide barcodes. **d**, Uniform manifold approximation and projection (UMAP) embedded in three dimensions, coloured by tissue annotation. Inset coloured by developmental time, matching colours in **a**,**b**. **e**, Cell type count mean (*x* axis) versus variance (*y* axis) for a subset of timepoints. The coefficient of variation (black line) and standard error (grey fill) for each cell type’s abundance is modelled using a generalized linear model with a gamma-distributed response. Cell types that vary significantly more than expected relative to the model are coloured in red (*P* < 0.05, maximum likelihood estimation). CNS, central nervous system; RBC, red blood cell; hindbrain NP, hindbrain neural progenitor (R7/8). Source Data

**Extended Data Fig. 2:**
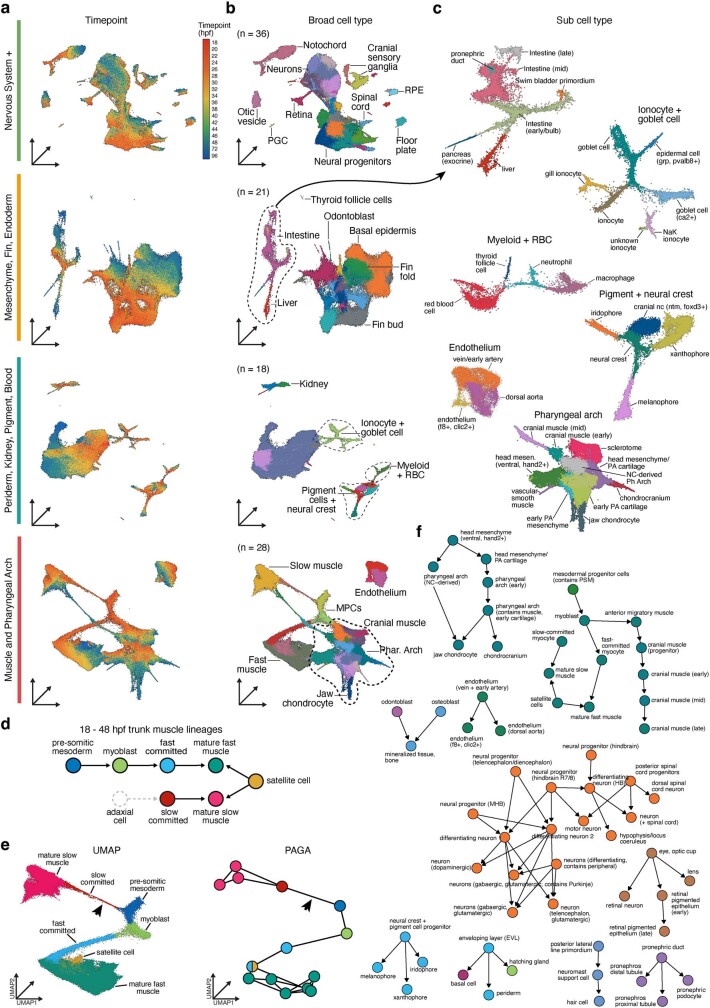
Hierarchical cell type annotations and lineage relationships. **a**, Sub-UMAP embeddings of the reference data, colored by timepoint. **b**, Sub-UMAP embeddings of each partition colored by cell type annotation with select cell types labeled. The number of broad cell types for that partition are listed. **c**, Select, tissue-specific regions of sub-UMAP embeddings with labels for all clusters corresponding to sub-cell type annotations. The number of sub-cell type annotations totals to 159, and the number for each group are as follows: periderm, kidney, pigment, blood = 43; mesenchyme, fin, endoderm = 27; nervous system = 53; muscle and pharyngeal arch = 43. **d**, True lineal relationships between trunk muscle cell types between 18 and 96 hpf. Adaxial cells and linkage to slow committed muscle shown as a dotted line to signify presence before 18 hpf (earliest collection). **e**, Transcriptional relationships between cells annotated as trunk muscle types in a UMAP dimensionality reduction plot (3D) and a graph made using the PAGA algorithm^74^. Arrows indicate connections that exist between transcriptional states. They do not necessarily represent true cell-lineage relationships. **f**, A graphical representation of cell types in our reference dataset harmonized with documented lineal relationships in ZFIN^75^.

We collected and labelled individual zebrafish embryos over 19 timepoints during embryonic and early larval development, spanning from 18 hpf, during late somitogenesis, with 2 h resolution until 48 hpf, then a 72 hpf timepoint and 96 hpf timepoint, a period marking the early larval stages (Fig. 1a). After quality control, our dataset included approximately 1.25 million cells from 1,223 barcoded individual embryos. At each timepoint, we collected between 48 and 140 embryos and amassed around 17,000–231,000 high-quality, single-nucleus transcriptomes per timepoint across four single-cell combinatorial indexing RNA sequencing (sci-RNA-seq3) experiments (Fig. 1b,c and Extended Data Fig. 1c–g). These data also integrated coherently with published zebrafish scRNA-seq data from earlier and overlapping timepoints, despite collection on different platforms (Extended Data Fig. 1h,i). Cell type identity was inferred by inspection of marker genes for each cluster, which were cross-referenced with annotated gene expression data from the zebrafish genome database, ZFIN. Overall, we hierarchically classified cells into 33 major tissues, 99 broad cell types and 156 cell subtypes (Fig. 1d, Extended Data Fig. 2a,b and Supplementary Table 2).

Given the continuity of many of our trajectories from one cell type to another, we sought to understand the lineal relationships reflected in our data (for example, the differentiation of mesodermal progenitors to fast muscle myocytes) (Extended Data Fig. 2d). However, inferring true lineage relationships from transcriptional similarity alone can be fraught^1^. For instance, pseudotime inference in the muscle trajectory suggested that slow and fast muscle cells share a common progenitor; however, slow muscle cells differentiate from an independent population of precursor cells that are present before 18 hpf (ref. ^10^), our earliest sampled timepoint (Extended Data Fig. 2e). To distinguish between bona fide lineage relationships and mere continuous transcriptional relationships across cell states in our atlas, we manually constructed a graph of documented lineage relationships, harmonized with our cell type annotations (Extended Data Fig. 2f).

**Extended Data Fig. 3:**
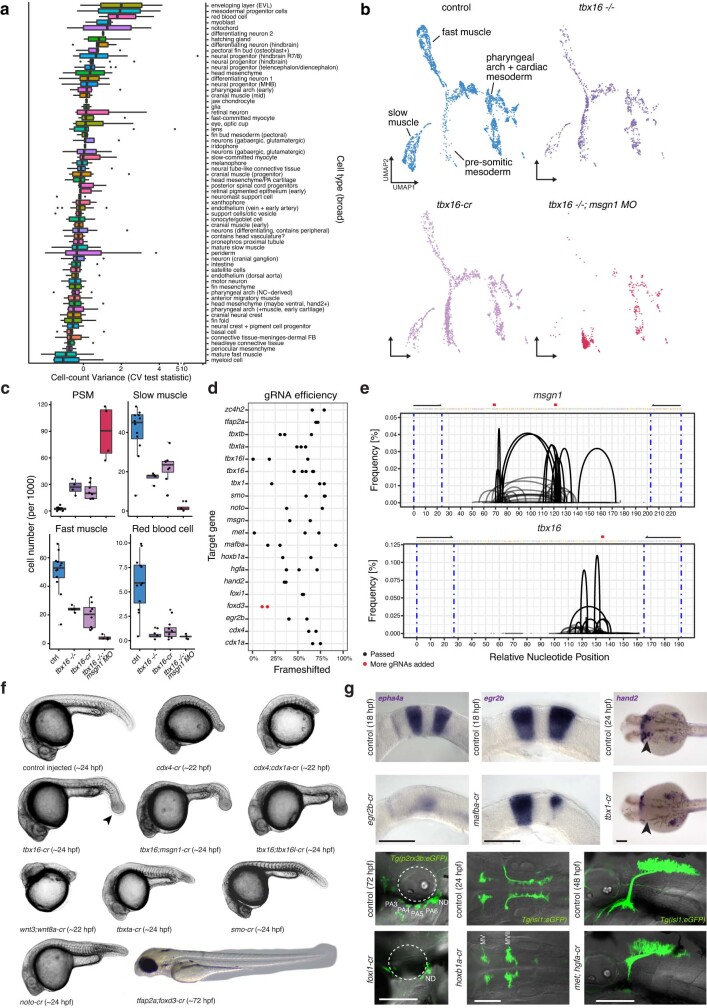
Proof-of-concept experiments, mutagenesis and phenotype validations. **a**, Cell count mean/variance relationships for all cell types per individual embryo, collapsed by time point and ranked by means. Mean-variance relationships are computed via beta-binomial modeling of the variance, followed by significance testing on the variance observed over the variance expected based on mean cell abundance. Colors denote different cell types. **b**, UMAP embedding of the mesodermal trajectory from whole-organism sci-RNA-seq (n = 5,929 cells; total n = 27,186 cells). Plots are faceted and colored by their perturbation (control-injected, n = 12 embryos; *tbx16* mutants, n = 4; *tbx16-crispant (cr)*, n = 8; and *tbx16 -/-*;*msgn1* morpholino (MO), n = 4). Major cell types are labeled in the first facet. **c**, Box plots of the size factor-normalized counts of each cell type recovered from individual embryos split out by perturbation. Cell types displayed are those predicted to have differential abundances in response to *tbx16* or *tbx16;msgn1* loss of function except periderm, which is unchanged. **d**, Percentage of frame shifted amplicons amplified from CRISPR-Cas9 edited zebrafish assessed via multiplex PCR. Extra guides were added for Foxd3 (red points) due to a low editing rate and the absence of the expected phenotype. **e**, Frequency of the cut sites detected within amplicons for Tbx16 and Msgn1. Black lines flanking the targeted region denote primers used for amplification of the amplicon. Protospacer adjacent motif displayed as a red box above the sequence. Mapping, analyses and plots deployed the ampliCan software package in R (v1.22.1)^64^. **f**, In addition to mutagenesis efficiency, gRNA sets were selected for their ability to generate phenotypes in F0 animals that resembled published null phenotypes. Representative images are labeled by their approximate developmental time and perturbation. **g**, For embryos where phenotypes were not apparent via whole mount, brightfield views, we evaluated the perturbation using appropriate transgenic lines or ISH. ISH target genes, perturbations, approximate timepoints, and anatomical landmarks are labeled (MV, trigeminal motor neurons; MVII, facial motor neurons; white dotted circle, ear; black arrow, posterior pharyngeal arches; PA#, pharyngeal arch number; ND, nodose ganglion). Scale bars, 100 µm. For all box plots, thick horizontal lines, medians; upper and lower box edges, first and third quartiles, respectively; whiskers, ±1.5× interquartile range; open circles, outliers. Source Data

Using our individual-resolved, whole-organism data, we were also able to estimate the variability of cell type abundances over developmental time. To estimate variance, we adapted a statistical framework commonly used to account for mean–variance relationships in sequencing experiments to model variability in cell abundances^11^. We found that most cell types vary in line with expectation given the nature of cell-count data, but we did see excess variance in some cell types. Cell types that were significantly variable (*P* < 0.05; Methods) include the enveloping layer (EVL), mesodermal progenitor cells (MPCs) and notochord cells at 20 hpf, and neural progenitor, optic cup, notochord and head mesenchyme cells at 36 hpf (Fig. 1e and Extended Data Fig. 3a). In addition to offering clues about the dynamic and transient nature of particular cell types, these variance estimates serve as important bases for our statistical assessment of perturbation-induced cell type abundance changes.

## Phenotyping embryos with scRNA-seq

Next, we used sci-Plex to label and measure single-cell profiles across time from developing zebrafish F0 knockouts (crispants) generated by CRISPR–Cas9 mutagenesis (Methods). We first compared individual crispants with mutants deficient for *tbx16* or both *tbx16* and *msgn1*, which have well-studied phenotypes at 24 hpf (ref. ^12^). Nearly all crispants were indistinguishable from stage-matched null mutants by gross morphology, displaying disorganized tail somite formation and the characteristic enlarged tail bud. We also looked for molecular or cellular differences between cells from knockout (crispant or null) to controls across 28 individual embryos. As previously documented^13–15^, both exhibited a marked loss of slow and fast muscle and accumulated MPCs, demonstrating the ability of our methodology to accurately pair genetic changes to loss of specific cell types (Extended Data Fig. 3b,c).

**Fig. 2:**
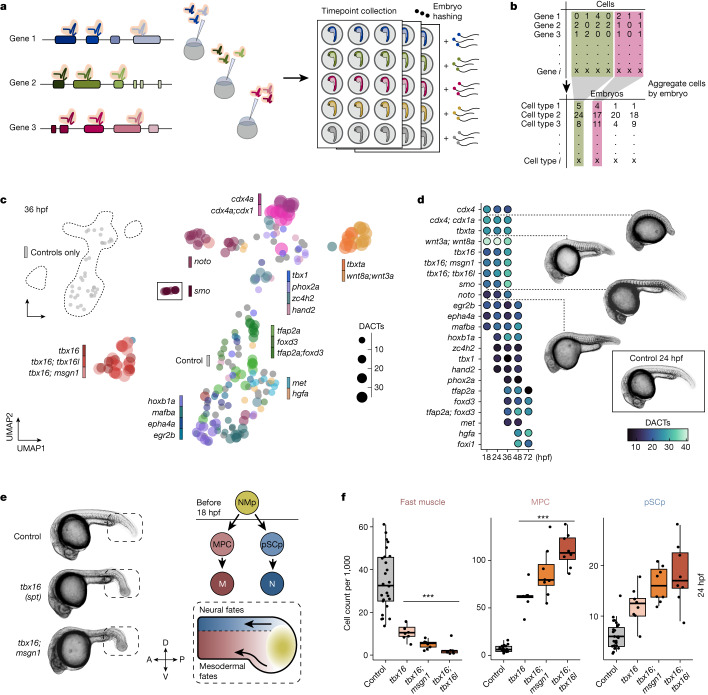
High-resolution phenotyping of crispant zebrafish embryos. **a**, A schematic of the experimental design. We designed two to three gRNAs across multiple exons and injected ribonucleoprotein complexes (RNPs) at the one-cell stage. Embryos were screened for phenotypes and dissociated in a 96-well plate before nuclei isolation, hashing and fixation. Partially created with BioRender.com. **b**, An individual by cell type matrix was constructed by tallying the number of each broad cell type recovered for each embryo. **c**, UMAP embedding of individual cell type composition data at 36 hpf. Embryos are coloured by genotype, and point size reflects the number of DACTs detected per genotype at 36 hpf. Control embryos are shown via inset (top left). *Smoothened* (*smo*) is shown as inset because it was distant to the other embryos. **d**, Heat map of DACT number for each perturbation and timepoint combination. Broad cell type annotation level (*n* = 99 total) was used, and abundance differences were deemed significantly different if *q* < 0.01 (beta-binomial regression). Images are representative siblings of collected embryos at 24–26 hpf. **e**, Representative images of control, *tbx16* and *tbx16*;*msgn1* crispants at 24 hpf, accompanied by a schematic of neuromesodermal differentiation in the tail bud (dashed box). NMps give rise to two anteriorly migrating lineages of cells: (1) MPCs and (2) pSCps, which give rise to somitic muscle (M) and spinal cord neurons (N), respectively. Compass denotes anatomical orientation: D, dorsal; V, ventral; A, anterior; P, posterior. **f**, Box plots of cell counts (per 1,000 and size-factor normalized) from individual embryos across selected cell types and genotypes 24 hpf (control *n* = 26, perturbed *n* = 8 embryos each). Thick horizontal lines show medians, box edges delineate first and third quartiles, respectively and whiskers extend to ±1.5× interquartile range. Significance (***q < 1 × 10^−4^, beta-binomial regression) relative to control embryos. Source Data

**Extended Data Fig. 4:**
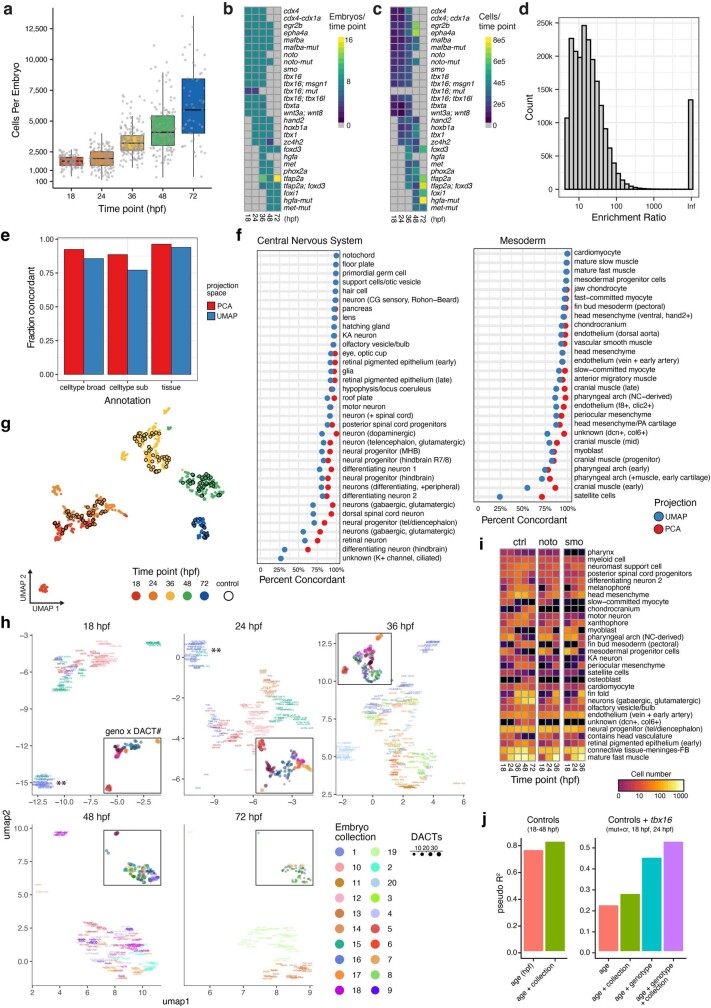
Analysis of transcriptional and compositional data across perturbations. **a**, Number of cells recovered per mutant embryo. Each gray point is an individual embryo that is summarized by the box plot. Previous estimates suggest that a 24 hpf embryo has ~25,000 cells^76^; based on this, we estimate a 5–10% recovery per embryo. Thick horizontal lines, medians; upper and lower box edges, first and third quartiles, respectively; whiskers, ±1.5× interquartile range. **b**, Heatmap displaying the number of embryos collected per perturbation × timepoint combination. **c**, Heatmap of the number of cells collected from each perturbation × timepoint combination. **d**, Hash-enrichment ratios for cells in the mutant dataset. Enrichment ratio was calculated as the ratio of top-ranked hash molecules observed in a cell divided by the second most abundant hash molecule after background subtraction. **e**, To test the accuracy of data projection, manually annotated wildtype reference cells were split 80:20. The 80 percent split was used as input for PCA, followed by UMAP. The 20 percent split was projected using the same transformations and labels were transferred in PCA space or UMAP space. Annotation labels were then transferred in either PCA space (red) or UMAP space (blue). Labels were deemed concordant if manual annotation matched the projected transfer annotation. **f**, Concordance of labels transferred in either PCA space (red) or UMAP space (blue) separated by broad cell type annotation. **g**, A UMAP plot where each point represents the cell type abundance composition (i.e. counts) for a single embryo, colored by collected timepoint. Rows of the input matrix constitute the union of all broad cell types, while columns are individual embryos. Perturbed embryos lack borders, and points with a black border is a control-injected or null wildtype sibling embryo. **h**, Low dimensional embryo embedding where embryos are colored by collection and the embedded text reflects the genotype. The plot is faceted by embryo age (hpf). **The location of the *tbx16* (mut or cr) embryos from separate collections but phenocopy one another in cell composition. Insets are the same embeddings colored by genotype and point size scaled to the number of DACTs for each genotype + timepoint. **i**, Heatmap depicting aggregated cell counts for select timepoint/cell type/mutant combination. Each box shows the number of cells (color) for a given timepoint (column) and broad cell type (row) combination. 30 cell types were sampled at random for display and three genotypes are shown control (left), *noto-cr* (middle), and *smo-cr* (right). **j**, Statistical assessment of variance in cell compositions with multinomial models, accounting for collection, genotype and age. In this case, embryo age and genotype explain the largest amount of variance with collection accounting for a small fraction. This may result from small shifts in stage or relative differences in fraction of cells lost during dissociation and fixation steps.

We then scaled up our approach to profile many different genetic perturbations spanning multiple timepoints during embryogenesis (Fig. 2a). In total, we targeted 23 genes or gene pairs involved in the development of either mesoderm (*cdx4*, *cdx1a*, *tbxta*, *tbx16*, *tbx16l*, *msgn1*, *wnt3a*, *wnt8a*, *noto*, *smo*, *tbx1*, *hand2*), central or peripheral nervous system (*egr2b*, *epha4a*, *hoxb1a*, *mafba*, *zc4h2*, *phox2a*, *foxi1*, *hgfa*, *met*) or neural crest lineages (*foxd3*, *tfap2a*) (Supplementary Table 3). We designed two to three guide RNAs (gRNAs) per gene and checked for editing efficiency at target regions via a sequencing-based assay (Extended Data Fig. 3d,e and Supplementary Table 4). A final set of gRNAs were chosen based on their ability to produce expected phenotypes in F0 knockouts without inducing non-specific cell death (Extended Data Fig. 3f,g). For each gene target, we collected eight embryos at an average of three of five timepoints that overlapped with the reference dataset: 18, 24, 36, 48 and 72 hpf. Altogether we profiled cells from 804 uniquely barcoded embryos across 98 conditions (including injection controls (*n* = 159), perturbations (*n* = 645) and multiple timepoints) and sequenced 2.7 million cells from a single sci-RNA-seq3 experiment and up to an estimated 10% of cells from each embryo (Fig. 2a and Extended Data Fig. 4a–d). Of these, the 600,000 or so cells from control-injected embryos did not display batch effects when co-embedded with our wild-type time-series reference, and they are included in the final reference dataset (Extended Data Fig. 1g).

To annotate cells by type for perturbed embryos and to facilitate cell type abundance analyses, we first projected the mutant data onto our reference atlas and then transferred annotations using a fast, approximate nearest-neighbour algorithm (Methods and Extended Data Fig. 4e,f). To assess perturbation-dependent cell type abundance changes, we transformed the data from a gene expression matrix into a cell type abundance matrix, effectively summarizing the number of each cell type observed within each embryo (Fig. 2b). After normalizing for the total cells recovered from each embryo, we performed dimensionality reduction to visualize these compositional data. Across the whole experiment, the primary source of variation in cell type proportions are embryo age and genotype, with marginal differences associated with embryo collection (Extended Data Fig. 4g–j). Within individual timepoints, perturbations with similar gross phenotypes readily grouped together; for example, loss of function for *tbxta* or *wnt3a*;*wnt8a*, all of which are important for maintenance of neuromesodermal progenitor cells (NMps)^16^. In contrast, knocking out the hedgehog receptor *smoothened (smo)* resulted in a distinct cell type composition at the whole-embryo scale, consistent with the widespread requirements of hedgehog signalling during development^17^ (Fig. 2c).

## Phenotyping with cell type compositions

**Extended Data Fig. 5:**
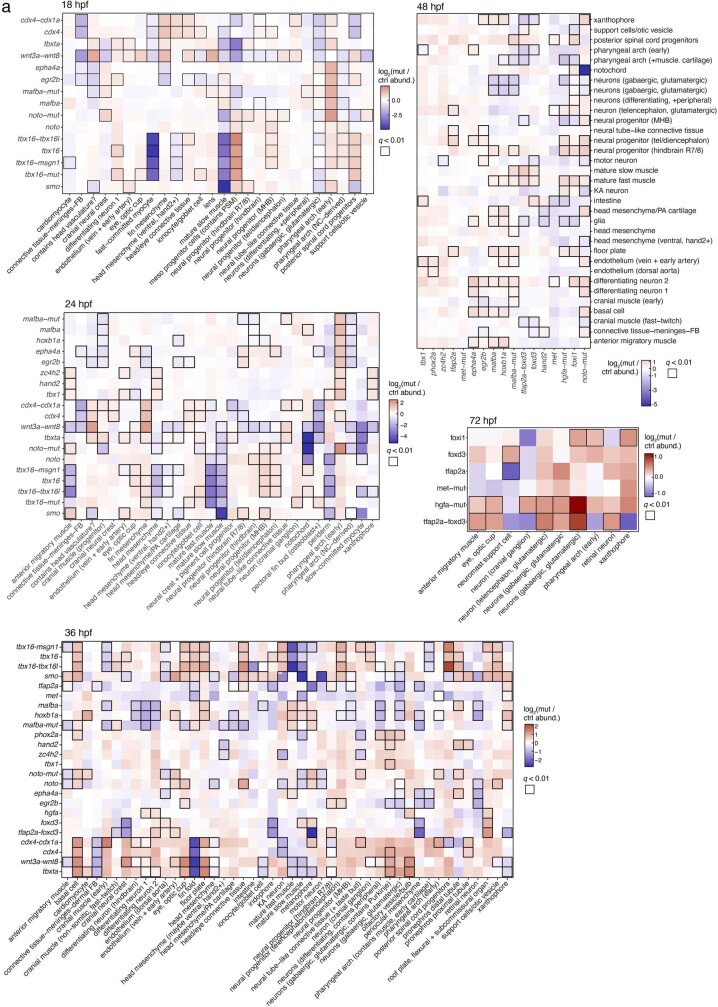
Differentially abundant cell types across all perturbations and timepoints. **a**, Heatmaps representing the log_2_ fold change in abundance of each perturbation relative to control-injected or wild type cells, for each broad cell type. Boxes indicate significant changes as determined using a beta-binomial regression modeling approach with multiple testing correction (*q-*value < 0.01, fold change > 1.5).

**Extended Data Fig. 6:**
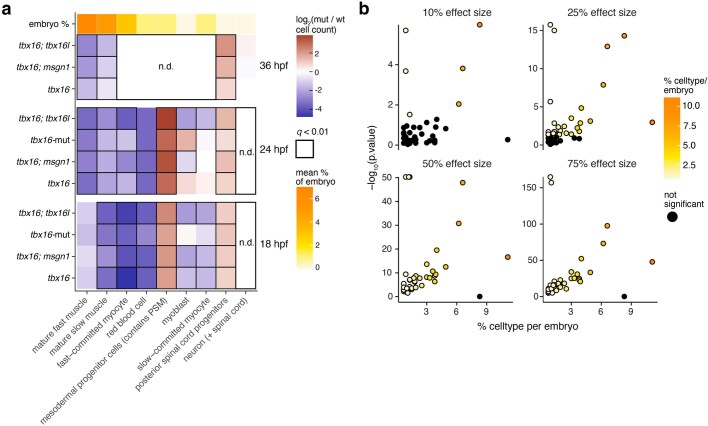
Differential cell type abundance across T-box groups in the mesoderm and spinal cord neurons. a, A subset of trunk muscle and spinal cord neuron cell types for each of four perturbations relative to control embryos at matched timepoints: *tbx16, tbx16; msgn1, tbx16; tbx16l*, and *tbx16*^*−/−*^. Black boxes indicate significance (*q* value < 0.01, beta-binomial regression with multiple testing correction; n.d. - no cells of this type detected at these stages). The mean percent of each cell type per whole embryo is represented by an additional color bar. **b**, A prediction of our ability to assign significance to cell type abundance changes across effect sizes and cell type proportions. The model is using the reference data as input and a Dirichlet multinomial distribution; *p*-values are assigned using our beta-binomial regression approach. Here, significant cell types are those with a *p* value < 0.05 (beta-binomial regression), points that fall below the threshold are black.

To systematically discern and rank all changes in cell type abundances across perturbations, we applied a beta-binomial regression model, which is well suited for assessing proportional changes in cell-count data^18^ (Methods). To robustly measure changes in cell type abundance, we collected replicate embryos (*n* = 8) for each perturbation/timepoint combination and compared them with stage-matched, control-injected embryos. Our analyses identified a range of significant differentially abundant cell types (DACTs) across the perturbations tested (Fig. 2d and Extended Data Fig. 5a). For example, crispant embryos for transcription factors that regulate the development of early somitic lineages—Tbx16, Msgn1 and Tbx16l (refs. ^13–15^)—exhibited both pronounced and subtle cell type abundance changes that were concordant between embryos (Extended Data Fig. 6a,b). This suite of transcription factors regulates differentiation of the NMp population that gives rise to MPCs and posterior spinal cord progenitors (pSCps) (Fig. 2e)^12^. Accumulation of stalled MPCs has been well characterized in *tbx16/msgn1* single and double mutants; however, the consequences to the pSCp lineage have not been examined. Our data show that within individual embryos, both MPC and pSCp lineages become progressively more abundant across single and double crispants (Fig. 2f). Thus, by examining whole transcriptomes, our data suggest that Tbx16, Tbx16l and Msgn1 interact to cooperatively control the differentiation of both mesodermal and neural progenitor cells from the NMp population and uncover putative sets of new target genes for these transcription factors in both cell populations.

## Perturbation-specific expression

**Extended Data Fig. 7:**
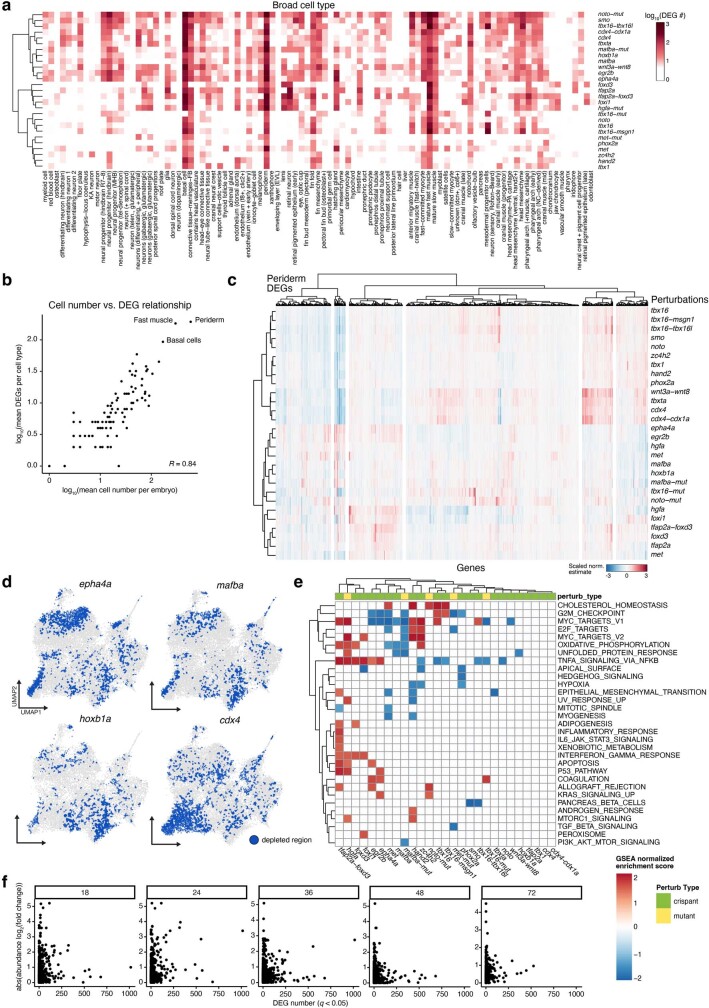
Transcriptional responses to genetic perturbations across targets and cell types. **a**, A heatmap displaying the number of differentially expressed genes (DEGs) (*q* < 0.05) for each broad cell type, across all perturbations. Numbers are displayed in log_10_(x + 1). **b**, A scatter plot comparing the mean number of DEGs (*q* < 0.05*)* for each cell type across perturbations to the mean number of cells per embryo (*pearson R* = 0.62). **c**, A heatmap displaying the normalized estimates from DEG testing in periderm cells across all perturbations (*q* < 0.05, n = 3206 genes). “Gene”-mut refers to null mutants (or -/-) rather than crispants. **d**, UMAP plots in which all neural progenitor cells are grey, and blue cells are control cells that are determined with the Getis–Ord test to have neighbors depleted for the perturbed cell type, termed “cold spots” for a selected set of perturbations known to affect hindbrain development. **e**, A heatmap displaying the normalized enrichment scores (NES) from a Gene Set Enrichment Analysis (GSEA)^72,77^ with the hallmark gene set on averaged, ranked estimates from differential expression testing across cell types for each perturbation. Only pathways with at least one significant enrichment are displayed (*p-adj* < 0.05, number of random gene sets with the same or larger value divided by the total number of generated sets, followed by multiple testing correction), and the color corresponds to the magnitude and direction of each significant enrichment; non-significant are white. Perturbations are annotated by whether they are null mutants (mutant) or F0 CRISPR/Cas9-injected (crispant). **f**, Scatter plots displaying the number of significant, differentially expressed genes between perturbed cells and control cells (y-axis), versus the absolute fold change in cell type abundance between perturbed and control (x-axis). Each point represents a unique cell type, perturbation pair, and plots are faceted by timepoint. Cell type specific, differentially expressed genes resultant to perturbation pairs are not associated with changes in cell type abundance.

To identify the transcriptional responses of each cell type to genetic perturbation, we performed differential gene expression tests to complement the differential abundance analysis. For each embryo, we combined cell data by type before testing (Methods). Pairwise differential gene expression tests between pseudo-bulked control and perturbed cells revealed an average of 1,470 differentially expressed genes (DEGs) for each perturbation, summed across all cell types (Extended Data Fig. 7a). Moreover, hierarchical clustering of DEGs highlighted that perturbations within a given genetic circuit induced common patterns of differential expression.

**Fig. 3:**
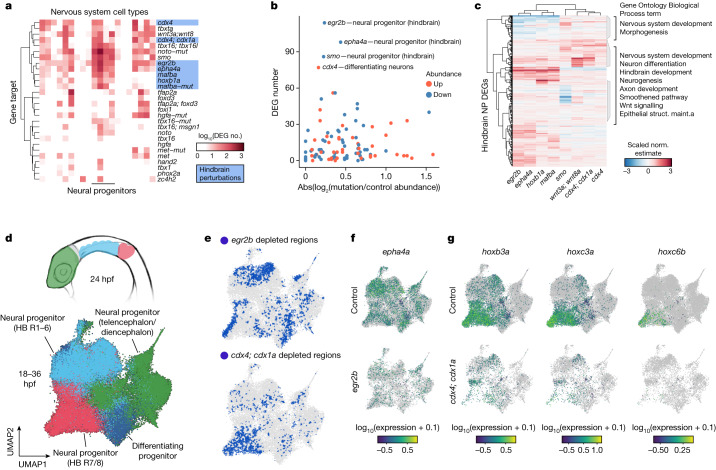
Systematic detection of DEGs and cell state changes across perturbations. **a**, Clustered heat map displaying the number of DEGs (displayed as log_10_(*x* + 1); *q* < 0.05) for neural cell types × all perturbation combinations. Hindbrain perturbations are highlighted in blue. **b**, The number of DEGs versus the absolute abundance change for hindbrain perturbation × neural cell type combinations. All collected timepoints are shown with abundance change direction denoted by colour. **c**, A heat map of the DEG coefficient estimates for hindbrain neural progenitor cells of embryos from eight perturbations affecting hindbrain development. Select significantly enriched Gene Ontology (Biological Process) terms are listed. Struct. maint., structural maintenance.  **d**, Diagram of a 24 hpf zebrafish (anterior, lateral view) (top), where anatomical regions are coloured to match the UMAP embedding (bottom) of subclustered neural progenitors from all perturbations and timepoints. **e**, UMAP embedding from **d**, where blue regions denote ‘cold spots’ (Getis–Ord test with multiple testing correction, *q* < 0.05): areas of the embedding where control cells are depleted for neighbours of the titled perturbation (*egr2b* above, *cdx4*;*cdx1a* below). **f**,**g**, UMAP plots in which cells are coloured by the expression of individual DEGs (*epha4a* (**f**), *hoxb3a*, *hoxc3a* or *hoxc6b* (**g**); *q* < 0.001) in controls, *egr2b* or *cdx4*;*cdx1a* crispant neural progenitor cells. Source Data

For example, we identified DEGs for neural progenitors for a suite of crispant perturbations that are known to affect neurogenesis (*cdx4*, *cdx1*, *wnt3a*, *wnt8a*, *mafba*, *hoxb1a*, *egr2b*, *smo* and *epha4a*) (Fig. 3a). While these perturbations did not result in robust cell type composition changes, we nevertheless uncovered many perturbation-induced DEGs (Extended Data Fig. 7f). Knocking out genes important for hindbrain neuron development—*egr2b*, *mafba, epha4a* and *hoxb1a* (ref. ^19^)—exemplified this phenomenon (Fig. 3b). Previous studies have revealed important roles for these factors in the segmentation and specification of neural progenitor cells in the hindbrain, but cell type-specific transcriptome-wide consequences of loss of function are unknown. When we compared DEGs for these perturbations, they form two major groups in accordance with known genetic interactions^20–22^. Moreover, the DEGs are enriched for biological processes and pathways involved in brain and nervous system development, offering new hypotheses for downstream effectors of our target genes (Fig. 3c).

Because neural progenitor cells at these stages have generally similar transcriptional programmes and do not form distinct boundaries in low dimensional space, we additionally sought to identify perturbation-dependent shifts in transcriptional states that were cluster agnostic (Fig. 3d). Here, we define the transcriptional states within the population of hindbrain progenitors by enrichment of gene expression in neural progenitors from rhombomeres 1–6 (for example, *egr2b*, *epha4a*, *mafba*), 7–8 (for example, *hoxa4a*), the diencephalon and telencephalon (for example, *vax1*, *vax2*, *fgfrl1a*) or differentiating neural progenitors (for exampled *elavl3*, *dla*, *dlc*, *ebf2*). We used the Getis–Ord test to identify regions of the reference UMAP embedding that were either enriched or depleted of perturbed cells in a co-embedded subset of the data (Methods). This analysis revealed distinct regions of the reference UMAP space that were depleted for perturbed hindbrain neural progenitor cells (Fig. 3e, Extended Data Fig. 7b). These regions corresponded to differential gene expression, such as a significant downregulation of *epha4a* expression in *egr2b* crispant neural progenitors, which is consistent with previous work^23^ (Fig. 3f). Previous studies of *cdx1* and *cdx4* identified functions during posterior mesoderm development, where they coordinate multiple pathways and activate *hox* gene expression^24^. Studies of zebrafish *cdx4*;*cdx1a* mutants also revealed the importance of these genes in hindbrain patterning^25^. Indeed, we find that three *hox* genes are significantly downregulated in *cdx4* and *cdx4*;*cdx1a* crispant neural progenitor cells (Fig. 3g). More broadly, our whole-embryo, single-cell measurements across time now enable a comprehensive view of candidate targets for these key transcription factors. These analyses highlight our ability to leverage individual-level transcriptome measurements to systematically evaluate perturbation-dependent transcriptional changes in each cell type and provide new hypotheses for functional studies.

## Dissecting the cranial sensory ganglia

**Fig. 4:**
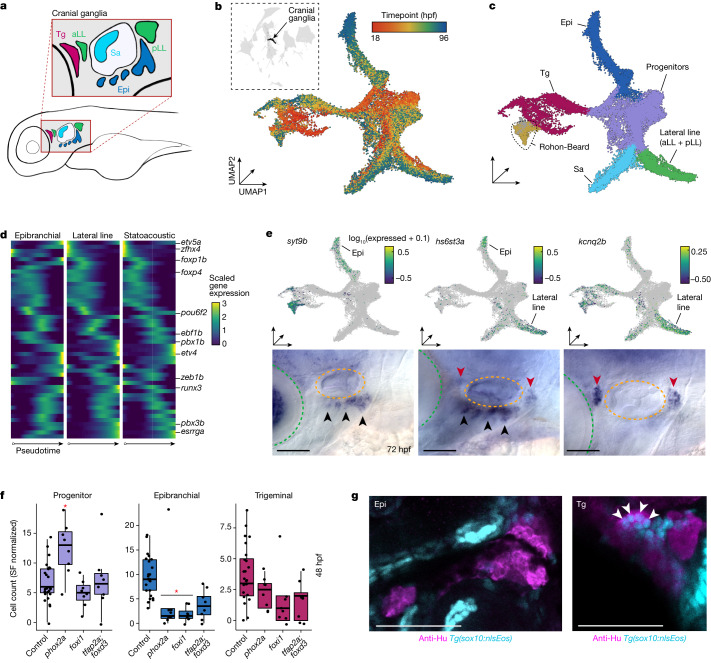
Whole-embryo phenotyping robustly captures effects in cranial sensory neurons. **a**, A lateral view diagram of the sensory cranial ganglia in an approximate 48 hpf zebrafish. Colours represent ganglia types: Tg, trigeminal ganglion; aLL, anterior lateral line ganglion; pLL, posterior lateral line; Epi, epibranchial ganglion; Sa, statoacoustic ganglion. **b**,**c**, Global UMAP embedding with cranial ganglia (*n* = 29,782 cells) and Rohon–Beard neurons in black (**b**, inset). Sub-UMAP of cranial ganglia coloured by timepoint (**b**) or cell type (**c**). Embeddings include wild-type cells and cells from perturbation experiments. **d**, Pseudotime heat maps of transcription factors enriched in one sensory ganglion trajectory branch. Genes listed on the *y* axis have previously identified roles in cranial ganglia development. **e**, UMAP expression plots (above) and lateral views of WISH at 72 hpf (below) for three genes specific to either the epibranchial ganglia (*syt9b*, left), lateral line ganglion (*kcnq2b*, right) or both (*hs6st3a*, centre). Lateral and anterior view, with eyes (green) and ears (orange) marked by dotted lines; arrowheads indicate epibranchial ganglia (black) or lateral line ganglia (red). **f**, Box plots of the sensory cranial ganglia cell type counts from individual embryos at 48 hpf *phox2a*, *foxi* and *tfap2a*;*foxd3* crispants. Significance is relative to control-injected embryos (*q < 0.05; beta-binomial regression with multiple testing correction; control *n* = 26; perturbed *n* = 8 embryos each; SF, size factor). Thick horizontal lines represent medians, box edges delineate first and third quartiles, respectively, and whiskers extend to ±1.5× interquartile range. **g**, A representative lateral view of cranial ganglia labelled with anti-HuC at 72 hpf. The Tg/aLL and Epi ganglia are visible in this maximum projection image. Single confocal slices of either the Tg/aLL or Epi ganglia labelled with anti-HuC and expressing *sox10:nlsEos* reveal subpopulations of neural crest-derived neurons in the Tg but not Epi ganglia. Arrowheads indicate co-labelled cells. Scale bars, 100 µm. Source Data

**Extended Data Fig. 8:**
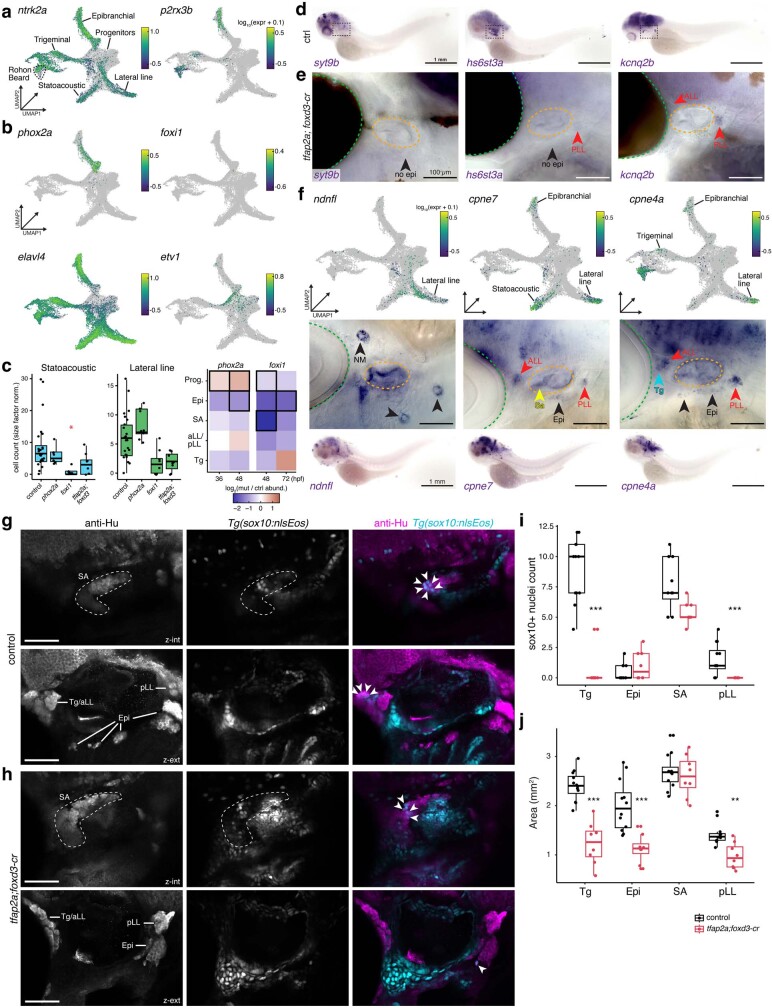
Subtype-specific gene expression and fate mapping in the cranial sensory ganglia. **a**, A UMAP plot where each cell is colored by its mean, normalized expression of neuronal or cranial ganglia markers: *ntrk2a, p2rx3b*. The legend scale bars reflect the expression bounds of each gene. **b**, A UMAP plot where each cell is colored by its mean, normalized expression of *phox2a*, *foxi1, elavl4, or etv1*. **c**, Cranial ganglia sensory neurons and their cell abundances (log2, size factor normalized) relative to control embryos at two timepoints for *phox2a* and *foxi1* crispants. Black squares indicate significance (*q* < 0.01, beta-binomial regression with multiple testing correction). **d**, Whole mount ISH at 72 hpf for *syt9b*, *hs6st3a*, and *kcnq2b*. The box represents the focus area imaged in order to resolve cranial ganglia expression. Scale bars, 1 mm. **e**, Representative whole mount ISH at 72 hpf in *tfap2a;foxd3* crispants. Eyes are dark since these embryos were not treated with the melanin-suppressing drug as in other in situs. Lack of staining compared to controls is noted. Scale bars, 100 µm. **f**, UMAP expression plots and corresponding ISH with a lateral head view and cranial ganglia labeled. Eye is marked by a green dotted line, ear is marked by an orange dotted line. (LL, lateral line ganglia; PLL, posterior lateral line ganglion; ALL, anterior lateral line ganglion; Tg, trigeminal ganglion; Epi, epibranchial ganglia; Sa; statoacoustic ganglion). Whole mount images of separate representative 72 hpf embryos are displayed below each higher magnification image. Scale bars, as marked. **g**, Single slices from a representative control animal at 72 hpf, displaying the statoacoustic (SA) ganglion, trigeminal/anterior lateral line (Tg/aLL), epibranchial (Epi), and posterior lateral line (pLL) ganglia. Anti-Hu and sox10:nlsEos are shown as single channels and merged. Sox10:nlsEos labels neural crest derivatives^57,78^. Co-labelled neurons are marked by white arrows. Scale bars, 50 µm; z-int, internal z slice; z-ext, external z slice. **h**, Single slices of a representative image from *tfap2a;foxd3*-*cr* embryo at 72 hpf. Neurons co-labeled with anti-Hu and sox10:nlsEos are indicated with white arrows. Scale bars, 50 µM. (***, *p-*value = 3.42e-8; two-sided Student’s *t*-test). **i**, Quantification of all Hu/sox10:nlsEos+ nuclei counts from from control (n = 12) at *tfap2a;foxd3-cr* (n = 8) animals at 72 hpf. **j**, Quantification of the HuC+ area in mm^2^ from Z-projected images from control (n = 12) at *tfap2a;foxd3-cr* (n = 8) animals at 72 hpf. (***, *p-*value = 2.55e-5 (Tg) or 1.4e-4 (Epi); **, *p-*value = 1.5e-3; two-sided Student’s *t*-test). Source Data

Specialized subsets of some cell types can express highly similar transcriptomes despite having distinct functions, lineage origins or anatomic locations^26,27^. Alternatively, cell types arising from distinct lineal origins can give rise to identically functioning cells^1,8,28^. Disentangling these unique scenarios may not be possible from snapshots of normal development, regardless of the resolution of the data. The cranial sensory neurons, which transmit information from the head, ear, heart and viscera, are examples of a cell type that has been difficult to study in zebrafish owing to their relatively low cellular abundance in the embryo, complex developmental history and a lack of known markers to distinguish their subtypes^29^. Despite their scarcity, we captured around 30,000 cranial sensory neurons (approximately 20 cells/embryo) contained within a single cluster, which formed four distinct branches upon subclustering. To identify whether these branches reflected placodal origins, neuronal function or something else, we manually compared branch-specific gene expression with published expression data. We concluded that, consistent with their distinct placodal origins, the branches represent the epibranchial, trigeminal, statoacoustic and lateral line cells, all radiating from a putative set of progenitors (Fig. 4a–c and Extended Data Fig. 8a).

We next sought to characterize the molecular differences between the subtypes of cranial sensory neurons and to identify the putative lineage-determining factors that distinguish them. Differential expression analysis identified 45 transcription factors that were expressed in the progenitors and just one of the daughter branches (Fig. 4d). This set of genes included some factors identified to regulate sensory neuron development^30^, but most have no previously reported role for these ganglia. To validate our cell type annotations and characterize new subtype markers, we additionally selected 11 terminally expressed genes to analyse by whole mount in situ hybridization (WISH). We were able to synthesize in situ hybridization (ISH) probes for 9 of these and found 8 that labelled the expected sensory ganglia at 72 hpf, establishing a new set of molecular markers for these subpopulations (Fig. 4e and Extended Data Fig. 8d).

To explore the genetic requirements of the cranial sensory ganglia, we disrupted two transcription factors that are important for their development: *foxi1* and *phox2a* (refs. ^31,32^). *Foxi1* is expressed early in development in placodal progenitor cells and is required broadly for proper differentiation of cranial ganglia neurons. *Phox2a* is required downstream of *foxi1* for development of epibranchial neurons, where it is specifically and robustly expressed (Extended Data Fig. 8b). Consistent with previous studies, we found that loss of *phox2a* led to a significant reduction of epibranchial neurons and an increase in progenitor cells, suggesting that these cells have stalled in a progenitor state. In *foxi1* crispants, progenitor cells and all four classes of cranial sensory ganglia were reduced, consistent with the early requirement of *foxi1* in placodal precursors of these lineages (Fig. 4f and Extended Data Fig. 8c).

Cranial sensory ganglia neurons have origins in the ectodermal placodes and embryonic neural crest, and the relative contributions from either origin are both ganglion and species dependent^33^. In zebrafish, the lineage contributions to each of the cranial ganglia are still unclear. Zebrafish cranial ganglia arise early in development predominantly from ectodermal placodes; later on, the neural crest contributes to trigeminal ganglia and potentially other classes^34,35^. In *tfap2a;foxd3* crispants, for which corresponding mutants lack nearly all neural crest derivatives^36^, we predicted that if neural crest cells contributed to specific ganglia, that we would detect corresponding decreases in cell abundance. We identified mean reductions (50–70%) in numbers of neurons of the trigeminal, epibranchial, statoacoustic and lateral line ganglia but not progenitors at 48 hpf (Fig. 4f and Extended Data Fig. 8c). Moreover, although their depletions did not reach statistical significance in any single timepoint, epibranchial and lateral line ganglia cells were consistently reduced across all three timepoints collected (36, 48 and 72 hpf). To more directly quantify neural crest contributions to epibranchial neurons, we performed lineage-tracing experiments which showed that they are not neural crest-derived at these developmental stages (Fig. 4g), as they are, to a certain extent, in other vertebrates^37,38^, and thus primarily depend on neural crest cells in a non-cell autonomous manner^39^. We did, however, detect a subset of trigeminal ganglion neurons that were neural crest-derived, consistent with previous fate-mapping results^34^. We additionally imaged the cranial ganglia in *foxd3;tfap2a* crispants and found a marked reduction in trigeminal and epibranchial ganglion size, consistent with our scRNA-seq results (Extended Data Fig. 8g–j). Taken together, our results demonstrate the potential of applying sci-Plex in conjunction with lineage-tracing tools to dissect the dependencies between cell types as the developmental programme unfolds.

## A shared notochord and cartilage programme

**Fig. 5:**
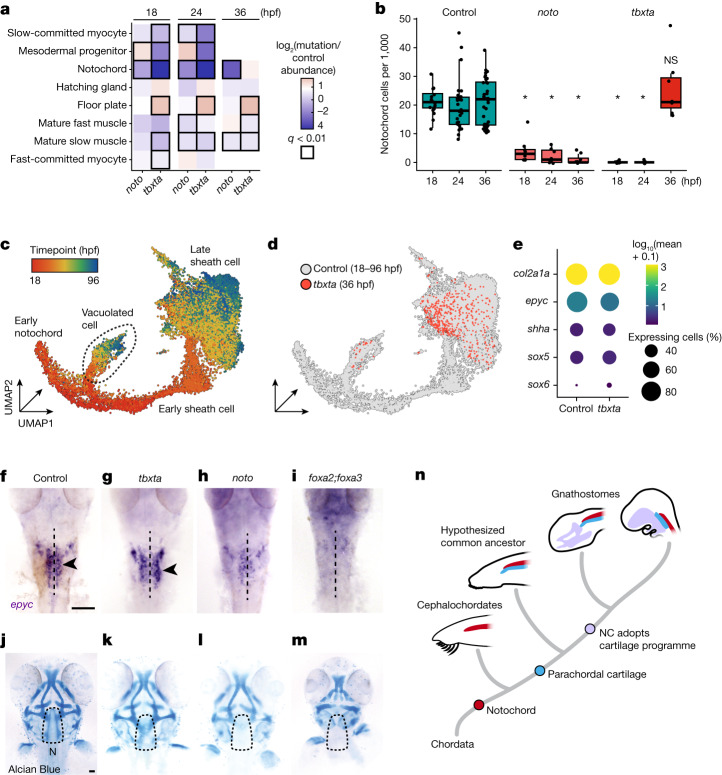
Tbxta and Noto perturbations uncover the genetic requirements of cranial cartilage development. **a**, Axial and paraxial mesodermal derivatives and their cell abundances relative to control embryos at three timepoints for *tbxta* and *noto* crispants. Black squares indicate significance (*q* < 0.01, beta-binomial regression). **b**, Box plots of notochord cell counts from individual embryos for controls, *noto* and *tbxta* crispants. Significance (*q < 1 × 10^−5^) is relative to wild-type control-injected embryos. Thick horizontal lines represent medians, box edges delineate first and third quartiles, respectively and whiskers extend to ±1.5× interquartile range. **c**, UMAP embedding of the notochord trajectory constructed with reference cells and *tbxta* cells. Cells are coloured by timepoint and are labelled by subtype annotation. **d**, UMAP embedding of notochord cells, coloured by genotype. **e**, A dotplot for a subset of genes that are expressed in notochord sheath, and in tbxta-independent cells, which are referred to as NLCs in the text. Colour represents mean normalized gene expression, and circle size indicates the percentage of notochord cells expressing the gene at 36 hpf. **f**–**i**, *epyc* ISH (36 hpf; dorsal, anterior view) in control (**f**), *tbxta* (**g**), *noto* (**h**) and *foxa2*;*foxa3* (**i**) crispants. The dashed line indicates the notochord, and parachordal cartilage cells in control and *tbxta* crispants are marked by black arrowheads. Scale bar, 100 µm. **j**–**m**, Alcian Blue staining of 72 hpf control (**j**), *tbxta* (**k**), *noto* (**l**) and *foxa2*;*foxa3* (**m**) crispants. Dashed outline surrounds the parachordal cartilage region. All *tbxta*, *noto* and *foxa2/foxa3* crispants lack a notochord. (N, notochord; dotted line surrounds the parachordal cartilage). Scale bar, 100 µm. **n**, A model depicting the hypothesized relationship between the notochord (NC) and cranial cartilage and bone elements over chordate evolution. Source Data

Because the notochord is the defining feature of chordates and serves critical structural and signalling roles in the vertebrate embryo^40^, we targeted two highly conserved transcription factors essential for its development: *noto* and *tbxta*/*brachyury*^41,42^. Our differential cell type abundance analyses largely reflected the expected phenotypes for *noto* and *tbxta*, for example, reduced slow muscle and notochord cells, and increased floorplate cells in *tbxta* crispants (Fig. 5a). In both *noto* and *tbxta* crispants, there is a near-complete loss of notochord cells at both 18 and 24 hpf. However, despite the absence of a visible notochord, we detected a near-complete recovery of putative notochord cells by 36 hpf in *tbxta* crispants (Fig. 5b).

**Extended Data Fig. 9:**
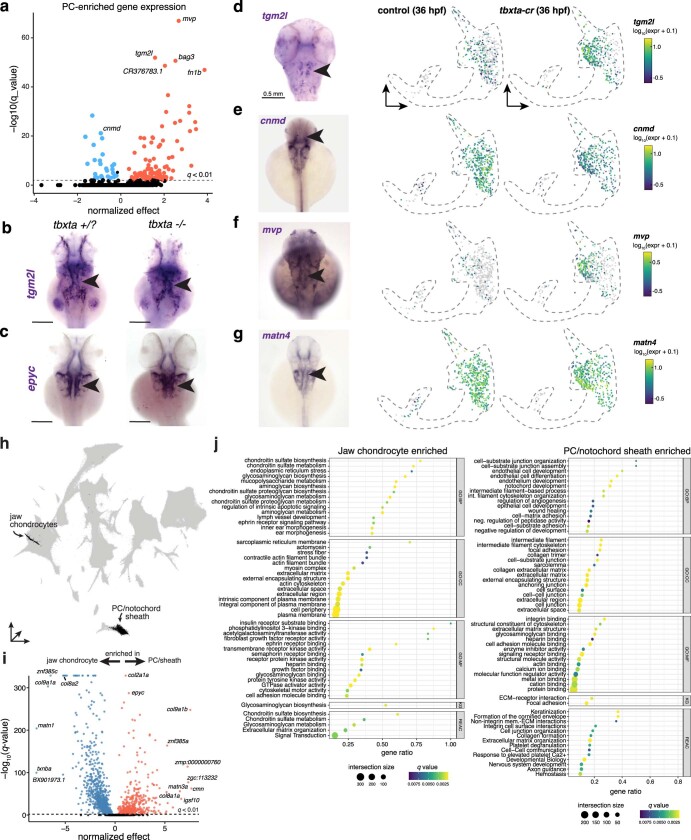
Notochord, parachordal and jaw cartilage transcriptome comparisons. **a**, A volcano plot representing the differentially expressed genes between *tbxta-cr* NLCs and control notochord sheath cells at 36 hpf. Genes enriched in *tbxta* cells are red and genes enriched in control cells are blue. Genes with a *q-*value > 0.01 (GLM with multiple testing correction) are black. The top five differentially expressed genes are labelled. **b**, *epyc in-situ* hybridization (36 hpf; dorsal, anterior view) in wildtype (or *tbxta* heterozygotes), and *tbxta* null mutants. **c**, *tgm2l* ISH at 48 hpf in wildtype (or *tbxta* heterozygotes) and *tbxta* mutants. Parachordal cartilage expression is indicated by a black arrowhead. **d**, Expression of *tgm2l* via in situ hybridization in parachordal in a wildtype embryo at 36 hpf (black arrowhead). A UMAP plot colored by the expression of *tgm2l* in the notochord of control and *tbxta*. *tgm2l* is enriched both in *tbxta* cells (*q* = 4.5e-61) relative to controls at 36 hpf and in the region of the UMAP predicted to be enriched for parachordal cartilage cells. **e**-**g**, Published *in-situ* hybridization stainings in prim 15 - prim 25 wildtype animals for *chondromodulin* (*cnmd), major vault protein* (*mvp)*, and *matrilin 4* (*matn4)*^73^ and UMAP plots from our study, from 36 hpf parachordal cartilage and notochord cells, colored by the expression of genes corresponding to the ISH and faceted by control and *tbxta-cr* cells (controls are downsampled to reflect the cell number in *tbxta-cr* samples). All scale bars, 0.5 mm. Scale bars are not available for ZFIN images^73^. **h**, A UMAP plot of the reference dataset, with the separate locations of jaw chondrocytes and parachordal cartilage/notochord sheath cells highlighted. **i**, A volcano plot displaying the differentially expressed genes between jaw chondrocyte and parachordal cartilage/notochord sheath cells (all post 36 hpf). Genes enriched in jaw chondrocytes are blue and genes enriched in parachordal cartilage/sheath are red (total n = 2132, *q* > 0.01). The top DEGs are labeled by name. x axis, normalized effect from the differential gene expression test; y axis, -log_10_-transformed *q*-value from differential gene expression test. **j**, The top 15 significant functional enrichment terms for the DEGs in jaw chondrocytes and parachordal cartilage/sheath. Gene ratio = intersection genes/genes in term; *q* < 0.01.

To investigate these unexpected cells (referred to as NLCs, notochord-like cells), we refined our annotations to distinguish the developmental trajectories of the two cell types that comprise the notochord: inner vacuolated cells and outer sheath cells (Fig. 5c). Vacuolated cells aid in embryonic axis elongation, while sheath cells form a surrounding epithelial layer that secretes a collagen-rich extracellular matrix around the notochord^43^. In *tbxta* crispants a majority of NLCs transcriptionally resembled maturing wild-type sheath cells (Fig. 5d). Comparison of NLCs relative to wild-type sheath cells revealed 157 genes with enriched expression, but all were still detected in both NLCs and wild-type sheath cells (*q* < 0.01, Extended Data Fig. 9a–e). At this point our mutant data had unmasked NLCs, a cryptic, sheath cell-like cell type (*epyc*+, *col2a1a*+, *shha*+) (Fig. 5e), arising between 24 and 36 hpf, despite the absence of a visible notochord.

To anatomically locate NLCs, we visualized the spatial localization of *epyc* expression using WISH. In control embryos, *epyc* is expressed weakly throughout the notochord and strongly in the parachordal cartilage, a conserved, mesodermally derived cartilage structure that later develops into the cranial base of the skull (Fig. 5f)^44,45^. Furthermore, another putative NLC marker revealed by our differential analysis, *tgm2l*, labelled parachordal cartilage cells but not notochord in wild-type embryos (Extended Data Fig. 9b,f). Consistent with the proposed similarities of the notochord sheath to cartilage^40^, we found that both cell types share the core conserved module of gene expression for cartilage formation (*sox5/6*, *col2a1a*), despite having thousands of DEGs (Fig. 5e and Extended Data Fig. 9h–j). Thus, the apparent and unexpected ‘recovery’ of notochord cells in *tbxta* crispants revealed that the NLCs, which are transcriptionally nearly indistinguishable from notochord sheath cells, are indeed parachordal cartilage cells.

**Extended Data Fig. 10:**
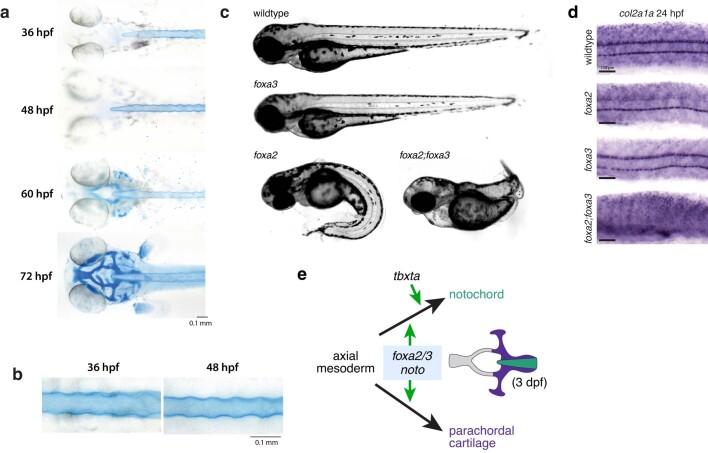
Anterior cartilage development and roles for *foxa2* and *foxa3* during notochord development. **a**, Anterior dorsal views of alcian blue-stained zebrafish embryos from 36 hpf to 72 hpf. **b**, Dorsal images of alcian blue-stained notochords at 36 and 48 hpf. **c**, Representative images of *foxa2*, *foxa3*, or *foxa2;foxa3* crispants at ~48 hpf. **d**, Trunk sections for *col2a1a* in situ hybridizations. No notochord cells are present in double *foxa2*;*foxa3* crispants. Scale bars, 100 µm. **e**, An updated model of the independent genetic requirements for PC and notochord development. Both structures derive from an early population of axial mesodermal progenitor cells. Cells that eventually become the notochord require *foxa2*, *foxa3*, *noto*, and *tbxta*, whereas *tbxta* is not required for the specification differentiation of axial mesodermal cells into the PC. (notochord is depicted in green and PC is depicted in purple at 3 dpf when PC is maturing).

The similarity between parachordal cartilage and notochord led us to wonder how their genetic requirements overlapped, so we visualized these cells in embryos lacking the lineage-determining factors *noto* and *tbxta*. In *tbxta* crispants and mutants, while notochord cells are missing, *epyc*+ early parachordal cartilage cells are present (Fig. 5f,g, Extended Data Fig. 9g). In *noto* mutants, *epyc* is weakly expressed by some cells in the posterior head, but these cells lack any organization around the midline. We next determined whether the *tbxta*-independent, early parachordal cartilage cells retained the ability to mature into chondrocytes by staining head cartilage at 72 hpf (Fig. 5j–l). The notochord sheath, the parachordal cartilage and the rest of the head cartilage is Alcian positive, supporting a common structural relationship between parachordal cartilage and notochord (Extended Data Fig. 10a,b). While Alcian-positive parachordal cartilage cells are present at 72 hpf in control and *tbxta* crispant embryos, posterior parachordal cartilage does not form in *noto*, consistent with the lack of *epyc*+ precursor cells (Fig. 5h). Thus, *tbxta* and *noto* have separate functions during parachordal cartilage and notochord development. To probe the earlier genetic requirements of these cells, we generated crispants for both *foxa2* and *foxa3*, two transcription factors with conserved roles during axial mesoderm specification. In mice, *foxa2* alone is required for notochord development, whereas in zebrafish, knockdown of *foxa2* and *foxa3* together leads to loss of all axial mesoderm derivatives^46,47^. We found that in the absence of both *foxa2* and *foxa3*, the notochord fails to develop, *epyc* + parachordal cartilage cells are missing, and no parachordal cartilage forms by 72 hpf (Fig. 5i,m and Extended Data Fig. 10c,d). Thus, while both the notochord and parachordal cartilage derive from the early embryonic *foxa2/3*-dependent axial mesoderm progenitor pool^48,49^, notochord development additionally requires *noto* and *tbxta*, whereas parachordal cartilage development only requires *noto* (Extended Data Fig. 10e). And although we sampled *tbxta* embryos at earlier timepoints (18 and 24 hpf), we did not identify any cells along the early notochord trajectory. This indicates that while differentiated parachordal cartilage cells share a transcriptional signature with notochord sheath cells, their progenitors are transcriptionally different and travel along separate differentiation trajectories. Together, these results show that parachordal cartilage and notochord fate divergence occurs early in the axial mesoderm, which is reflected by the different genetic requirements of the parachordal cartilage and the notochord.

## Discussion

Here we present a new approach (whole-organism labelling) and dataset, termed ZSCAPE, for systematically analysing the impact of genetic perturbations on each cell type in thousands of developing zebrafish at single-cell resolution. Critically, our workflow’s costs are dominated by sequencing, so profiling cells from many samples is only marginally more expensive than profiling a similar number of cells from few specimens. We first established an individual-resolved reference atlas of zebrafish development. Our data fill a gap in existing zebrafish atlas datasets^7–9^, providing a single-cell dataset comprising 19 timepoints from 18 to 48 hpf. This developmental period features the differentiation of diverse cell types and tissues throughout the organism, and the accompanying cell type annotations reflect this richness (33 major tissues, 99 broad cell types and 156 cell subtypes). Because the atlas is derived from cells from over 1,000 individually barcoded animals, we used it to quantify variability in proportions of each cell type in the embryo.

Although forward genetic screens have revealed hundreds of genes required for zebrafish development, the field’s inventory of cell types that depend on each is incomplete. We studied 23 genes with phenotypes ranging from well characterized (for example, *tbxta* and *tbx16*) to largely unexplored (*epha4a*). Our experiments expand these genotype–phenotype mappings embryo-wide by describing the molecular and cellular consequences of each perturbation. We collected 2.7 million single-cell transcriptomes from 804 mutant or crispant embryos across 98 conditions in a single sequencing experiment. The unprecedented depth of replication in the experiment, with at least 16 embryos per genotype, afforded statistical power to comprehensively detect gains and losses in the abundance of both common and rare cell types throughout the embryo. For example, we dissected the molecular signatures of the sensory cranial ganglia neurons and their precursors, which are a diverse set of cells that together comprise fewer than 1% of the embryo. Sequencing whole crispants focused our use of more conventional genetic tools on phenotypes in specific cell types and tissues of interest without requiring complex reporter systems or other means of purifying cells of interest, a priori. Our experiments also expanded phenotypes for even intensively studied genes. For example, we detected stalled spinal cord progenitor cells in *tbx16, tbx16-msgn* or *tbx16-tbx16l*, suggesting a previously unappreciated dependency on these genes. Moreover, by integrating cell type-specific molecular phenotypes with morphological and spatial information in *tbxta* and *noto* mutants, both of which fail to develop notochords, we identified the parachordal cartilage as transcriptionally indistinguishable from notochord sheath cells. This revealed independent genetic requirements for these two cell types, a finding that provides new clues about the origins of the vertebrate skull.

The high degree of transcriptional similarity and differing genetic requirements of parachordal cartilage cells (‘true cartilage’) and notochord sheath cells (‘cartilage like’)^50^ offers clues into the evolutionary origin of vertebrate cranial skeletons. While it is now clear that much of the anterior head cartilage is neural crest derived, the evolutionary origin of the ancient mesodermal head cartilage, which produces the posterior skull, is unknown^44,45^. Based on the shared location, gene expression and transcriptional regulation of the progenitors for parachordal cartilage and notochord, we speculate that the cartilage-like notochord cells are the direct precursors to skeletal cranial elements in the vertebrate lineage. Thus, we suggest that as creatures evolved from an amphioxus-like vertebrate ancestor, some of the embryonic anterior notochord cells split to form the parachordal cartilage just lateral to the notochord, which allowed the development of more complex mesodermal cartilage structures. Later, these joined with neural crest-derived cartilage to form the modern vertebrate skull (Fig. 5n)^51^. These findings highlight the promise of high-resolution molecular phenotyping to deepen our understanding of the relationship between gene expression and genetic networks, facilitating new hypotheses about the evolutionary origins of individual cell types.

Our method is not without limitations for future research to address. First, while we are well powered to detect changes in certain lowly abundant cell types, the statistical power required is still dependent on the magnitude of the effect and the number of replicates profiled. Additionally, while observing phenotypes in a whole-organism context offers advantages, profiling larger organisms that may contain billions to trillions of cells may be infeasible. Nevertheless, in a concurrently published study in this issue, a similar approach is taken in the mouse^52^, such that replicate embryos of multiple genotypes can be profiled at single-cell resolution. Finally, while we assessed mutagenesis efficiency at the whole-embryo level before single-cell sequencing, low levels of mosaicism in F0 crispants are a concern, especially when this approach is used for morphogens or other secreted factors where a small amount of mosaicism may be sufficient to rescue a mutant phenotype. An ideal assay would capture both the single-cell transcriptome and the perturbed genetic allele, allowing for the interpretation of perturbations with no apparent phenotype.

Looking forward, we anticipate that using single-cell sequencing to measure the consequences of many embryos perturbed in different ways will open up rich opportunities for developmental genetics. Sequencing many embryos in each genotype or treatment group enables one to use tools from statistical inference that are unavailable when analysing only a handful of specimens. In related work, we applied sci-Plex in hundreds of embryos to quantify cell type-specific responses to increased temperature during zebrafish development^53^. We expect that the data presented here will inspire new computational tools aimed at reconstructing gene networks, clarifying cell-lineage relationships and illuminating new mechanisms of robustness, as all these areas of computational biology are rich with statistical challenges posed by inherent variability, missing data, feedback and hypothesis testing. Moreover, cell-hashing techniques are compatible with other single-cell sequencing modalities, so in principle, phenotyping could be conducted at the level of chromatin, spatial readouts of morphology or the proteome. As our field accumulates a catalogue of whole-embryo, single-cell transcriptional phenotypes, the potential for discovering mechanisms through which the vertebrate genome controls development using computational and statistical tools will only grow.

## Methods

### Animal rearing, staging and stocks

Staging followed^54^ and fish were maintained at around 28.5 °C under 14:10 light:dark cycles. Fish stocks used were: wild-type AB, *noto*^*n1*^ (ref. ^41^),* tbx16*^*b104*^ (ref. ^13^), Tg(*isl1*:*gfp*)^*rw0*^, Tg(*p2rx3*:*gfp*)^*sl1*^, *mafba*^*b337*^ (ref. ^55^), *hgfa*^*fh528*^, *met*^*fh533*^ (ref. ^56^) and Tg(*sox10*:*nlsEos*)^*w18*^ (ref. ^57^). Fish were anaesthetized before imaging or dissociation with MS222 and euthanized by overdose of MS222. All procedures involving live animals followed federal, state and local guidelines for humane treatment and protocols approved by Institutional Animal Care and Use Committees of the University of Washington and the Fred Hutchinson Cancer Center.

### Image analysis

Confocal image stacks of the cranial ganglia from individual fish were processed equally, and cell counts were made in ImageJ by comparing nuclear and cytoplasmic fluorescence in parallel. Area measurements of cranial ganglia were done in ImageJ by applying manual bounds to maximum projections of HuC staining, which labels the cell bodies of neurons. Images were counted and measured blindly.

### In situ hybridization, immunohistochemistry and labelling

Alkaline phosphatase ISH was performed using standard conditions^58^. We used the following riboprobes and antibodies: *col2a1a*, *tgm2l*, *epyc*, *syt9b*, *hs6st3a*, *kcnq2b*, *nfl*, *cpne7*, *cpne4a* (all this study), *hand2*^59^, *epha4a*^60^, *egr2b*^61^, anti-HuC/D (mouse monoclonal antibody, Thermo Fisher, catalogue no. 16A11, 1:750), Goat anti-Mouse IgG Alexa Fluor 647 (Thermo Fisher, catalogue no. A21236, 1:400). For all immunohistochemistry, embryos were collected at reported stages, anaesthetized with MS222 (10 mg ml^−1^ in buffered embryo medium; Sigma-Aldrich) and fixed in 4% paraformaldehyde overnight at 4 °C. Antibody staining was performed as previously described^62^. Alcian blue staining followed an online procedure (The Society for Developmental Biology Online Short Course, Zebrafish Alcian Blue), except that embryos were raised in 1-phenyl-2-thiourea (MilliporeSigma, catalogue no. P7629) to suppress pigment formation rather than bleaching. After staining, the embryos were moved into 70% glycerol, the yolk was removed and the embryos were flat-mounted under a coverslip. Alcian Blue-stained embryos and ISH embryos were imaged on a Nikon AZ100 microscope. For confocal images in Fig. 4 and Extended Data Fig. 8g–j, imaging was performed on a Zeiss LSM 880 laser scanning confocal microscope with a ×10 Plan-Apochromat 0.45 objective and an Airyscan super-resolution module, and Zen Black acquisition software (Zeiss). Fish were imaged for Alexa Fluor 594 (anti-Hu) with a 561 nm laser and for nuclear-Eos with a 488 nm laser. A step size of approximately 1.5 µm was used to acquire 40–80 slices, depending on the sample. To increase signal-to-noise ratio and resolution, acquired images were processed by two-dimensional Airyscan filter strength 7.0 with Zen Black software. Images were opened in Fiji as .czi files for nuclei counts across conditions. For confocal imaging in Extended Data Fig. 3g, embryos were anaesthetized with MS222 and mounted in 1% low-melt agarose on a coverslip and imaged on an LSM700 inverted confocal microscope at ×20 magnification.

### CRISPR–Cas9 mutagenesis in zebrafish embryos

gRNAs were designed using either the Integrated DNA Technologies (IDT) or CRISPOR^63^ online tools. gRNA and RNP preparation closely follow a recently published protocol for efficient CRISPR–Cas9 mutagenesis in zebrafish^6^. Briefly, gRNAs were synthesized as crispr RNAs (crRNAs, IDT), and a 50 µmol crRNA:trans-activating crispr RNA (tracrRNA) duplex was generated by mixing equal parts of 100 µmol stocks. Cas9 protein (Alt-R S.p. Cas9 nuclease, v.3, IDT) was diluted to a 25 µmol stock solution in 20 nmol HEPES-NaOH (pH 7.5), 350 mmol KCl, 20% glycerol. The RNP complex mixture was prepared fresh for each injection by combining 1 µl 25 µmol crRNA:tracrRNA duplex (with equal parts each gRNA per gene target), 1 µl of 25 µmol Cas9 Protein and 3 µl nuclease-free water. Before injection, the RNP complex solution was incubated for 5 min at 37 °C and then kept at room temperature. Approximately 1–2 nl was injected into the cytoplasm of one-cell-stage embryos.

### Genotyping

At 2 days after CRISPR–Cas9 RNP injections (48 hpf), pools of five F0-injected embryos for each gRNA set were lysed in 100 μl alkaline lysis buffer (25 mmol NaOH, 0.2 mmol ethylene-diamine-tetra-acetic acid (EDTA)) and heated at 95 °C for 30 min. The solution was neutralized by an equal volume of neutralization buffer (40 mmol Tris-HCl, pH 5.0). Rhamp-seq primers were designed using the Rhamp-seq IDT design tool. Rhamp-seq primers were reconstituted in low-Tris-EDTA buffer (10 mmol Tris/HCl ph 7.4, 0.1 mmol EDTA) to a final concentration of 10 μmol. These primers were then mixed in four pools as specified by the IDT design tool (Pool1-FWD, Pool1-REV, Pool2-FWD and Pool2-REV). Each primer in these pools was mixed such that the primer’s final concentration in the pool was 0.25 μmol. Genotyping PCRs for each crispant were performed using 5 μl of 4× Rhamp-seq Master Mix 1 (IDT), 2 μl of FWD pool, 2 μl of REV pool and 11 μl of gDNA template. Twenty cycles of PCR were performed using the following thermocycler programme:

1. 95 °C for 10 min
2. 95 °C for 15 s
3. 61 °C for 4 min
4. Return to step 2 for 10 cycles total
5. 99.5 °C for 15 min

Following amplification, PCR products were purified using a 1.5× SPRI bead cleanup (Beckman Coulter, catalogue no. A63880) and eluted in 15 μl low-Tris-EDTA buffer. Index PCR was performed using 5 μl of 4× Rhamp-seq Master Mix 2, 2 μl of Indexing PCR primer (i5), 2 μl of Indexing PCR primer (i7) and 11 μl of purified PCR product. An additional 20 cycles of index PCR were then performed using the following thermocycler programme:

1. 95 °C for 10 min
2. 95 °C for 15 s
3. 60 °C for 30 s
4. 72 °C for 30 s
5. Return to step 2 for 20 cycles total
6. 72 °C for 1 min

After the index PCR, sequencing libraries were pooled, purified with a 1× SPRI bead cleanup and sequenced on the Illumina MiSeq 600 cycle kit with 2 × 300 cycle paired-end reads. Reads were analysed using the ampliCan software package with default settings and standard vignette workflow^64^.

### Preparation of barcoded nuclei

Individual zebrafish embryos (18 to 96 hpf) were manually dechorionated with forceps and transferred to a 10 cm petri dish containing 1× TrypLE (Thermo Fisher, catalogue no. 12604013). Using a widebore tip, embryos were transferred, one by one, into separate wells of a 96-well V-bottom plate containing 75 μl of 1× TrypLE (Thermo Fisher, catalogue no. 12604013) + 2 mg ml^−^^1^ Collagenase P (MilliporeSigma, catalogue no. 11213865001). Embryos were then dissociated by 10 strokes of manual trituration at 30 °C once every 5 min. Dissociation continued until no visible chunks were present under a dissecting scope, which took between 20 and 40 min depending on embryo stage (for example, 20 min for 18 hpf and 40 min for 72 hpf). Stop solution (1× Dubecco’s phosphate-buffered saline (dPBS) (Thermo Fisher catalogue no. 10010023), 5% FBS (Thermo Fisher catalogue no. A4736401)) was then added to each well to quench the proteases. Cells were then spun down at 600*g* for 5 min. Cells were then re-suspended in 200 μl in cold dPBS and spun down again. After rinsing, the supernatant was removed fully and cells were re-suspended in 50 μl of cold lysis buffer (10 mmol Tris/HCl pH 7.4, 10 mmol NaCl, 3 mmol MgCl_2_, 0.1% IGEPAL, 1% (v/v) SuperaseIn RNase Inhibitor (20 U µl^−1^, Ambion), 1% (v/v) BSA (20 mg ml^−1^, NEB)) + 5 μl of hash oligonucleotide (10 µmol, IDT) and incubated for 3 min on ice. Following lysis, 200 μl of ice cold, 5% fixation buffer (5% paraformaldehyde (EMS, catalogue no. 50-980-493), 1.25× dPBS) was added to each well. After an additional round of mixing, nuclei were fixed on ice for 15 min. All wells were then pooled together in a 15 ml conical tube and spun down for 15 min at 750*g*. Supernatant was decanted and cells rinsed in 2 ml of cold NBB (Nuclei Buffer + BSA: 10 mmol Tris/HCl pH 7.4, 10 mmol NaCl, 3 mmol MgCl_2_, 1% (v/v) BSA, 1% (v/v) SuperaseIn RNase Inhibitor) at 750*g* for 6 min. Supernatant was then carefully aspirated, and the nuclei were re-suspended in 1 ml of NBB and flash frozen in LN_2_ and stored at −80 °C.

### sci-RNA-seq3 library construction

The fixed nuclei were processed similarly to the published sci-RNA-seq3 protocol^2^ with some modifications. Briefly, frozen, paraformaldehyde-fixed nuclei were thawed, centrifuged at 750*g* for 6 min and incubated with 500 μl NBB (see previous) including 0.2% (v/v) Triton X-100 for 3 min on ice. Cells were pelleted and re-suspended in 400 μl NBB. The cell suspension was sonicated on low speed for 12 s (Diagenode, Bioruptor Plus). Cells were then pelleted at 750*g* for 5 min before re-suspension in NB + dNTPs. The subsequent steps were similar to the original sci-RNA-seq3 protocol (with paraformaldehyde-fixed nuclei) with some modifications, and a detailed, step-by-step protocol is available in the Supplementary Protocol.

### Sequencing, read processing and cell filtering

Libraries were sequenced on either an Illumina NextSeq 500 (High Output 75 cycle kit), Nextseq 2000 (P2 100 cycle kit) or Novaseq 6000 (S4 200 cycle kit) with sequencing chemistries compatible with library construction and kit specifications. Standard chemistry: Index 1, 10 bp; Index 2, 10 bp; Read 1, 34 bp; Read 2, remaining cycles (more than 45 bp). Read alignment and gene-count matrix generation were performed using the Brotman Baty Institute pipelines for sci-RNA-seq3 (https://github.com/bbi-lab/bbi-dmux; https://github.com/bbi-lab/bbi-sci). After the single-cell gene-count matrix was generated, lower  unique molecular identifier (UMI) thresholds were determined for each experiment  (from 100–250), followed by removal of cells with UMIs greater than four standard deviations from the mean. For mitochondrial signatures, we aggregated all reads from the mitochondrial chromosome, and cells with more than 25% mitochondrial reads were removed. Each cell was assigned to a specific zebrafish embryo based on the enrichment of a single hash oligonucleotide, as described previously^5^. Enrichment cutoffs were set manually based on the distribution of enrichment ratios (Supplementary Table 1). Removing cells with low hash-enrichment ratios eradicated most multiplets^5^. Additional clusters of multiplets not removed using this procedure were manually inspected for marker genes and removed.

### scRNA-seq analysis

After RNA and hash-quality filtering, data were processed using the Monocle3 (v.1.3.1) workflow defaults except where specified: *estimate_size_factors()*, *detect_genes(min_expr* = *0.1)*, *preprocess_cds()* with 100 principal components (using all genes) for whole-embryo and 50 principal components for subsets, *align_cds(residual_model_formula_str* = *“~log10(n.umi)”)*, *reduce_dimension(max_components* = *3, preprocess_method* = *‘Aligned’)* and finally, *cluster_cells (resolution* = *1e-4)*.

### Hierarchical annotation and subclustering

To build maps where cluster annotations corresponded broadly to cell types, we first split the global reference dataset into four major groups that each contained either the epidermis, muscle, central nervous system neurons or mesenchyme cells, along with other nearby cell types. Each of these groups was re-processed, embedded in three dimensions with UMAP and subclustered. Cluster resolution was optimized such that major groups were composed of 30–70 clusters that qualitatively represented the transcriptional diversity in a given set. Clusters were then assigned annotations based on the expression of marker genes (using the top_markers function, significance assessed using a two-sided likelihood ratio test with multiple comparisons adjusted; Supplementary Table 8) based on literature by an unsupervised signature-scoring method using anatomical-term gene lists from the ZFIN database (zfin.org). With the exception of a few additional subclustering examples (that is, the cranial ganglia), each cluster was assigned on ‘cell_type_sub’ annotation. These subtype annotations were manually merged into ‘cell_type_broad’ classifications based on cluster proximity or cell type functional groupings. We further merged these annotations into ‘tissue’ groups based on whether broad cell types together composed a broader tissue. Finally, we designated each cell type into a ‘germ_layer’ group based on the known germ layer of origin.

### Individual-level composition analysis

After cell type annotation, counts per cell type were summarized per embryo to generate an embryo × cell type matrix. Embryo composition size factors were calculated independently for each timepoint. The embryo × cell type matrix was stored as a cell_data_set object, allowing for preprocessing (PCA) and dimensionality reduction (UMAP) using the standard Monocle3 workflow.

### Query dataset projection and label transfer

The PCA rotation matrix, batch-correction linear model and UMAP transformation were computed and saved during the processing of the reference dataset. This computation was done on two levels: first, with all combined reference cells (global reference space), and second, in each of four subgroups (subreference space). The query dataset was first projected into the global reference space using the following procedure: the PCA rotation matrix, which contains the coefficients to transform gene expression values into PCA loadings, was applied to the query dataset. The batch-correction model was then applied to the resulting query PCA matrix to remove the effects of the UMI count. Finally, the reference-calculated UMAP transformation was applied to the batch-adjusted PCA loadings to project the query data into the stable reference coordinate space. This procedure is similar to the procedure used in Andreatta et al.^65^ One of four major subgroup labels was transferred (mesoderm, mesenchyme-fin, periderm, CNS) using the majority label of its annotated nearest neighbours (*k* = 10). Nearest neighbours were calculated using annoy, a fast, approximate nearest-neighbour algorithm (https://github.com/spotify/annoy, v.0.0.20). The query dataset was split into four subgroups based on these assigned major group labels. Each query subgroup was projected into the subreference spaces using the corresponding saved PCA, batch correction and UMAP transformation models using the same projection procedure. Finer resolution annotations (germ layer, tissue, broad cell type, subcell type) were transferred in this subspace using the majority vote of reference neighbours (*k* = 10).

### Differential expression testing

Before differential expression testing, expression values were aggregated for each embryo across each cell type into ‘pseudo-cells’. We pooled embryos across timepoints and only compared embryos from the same sets of timepoints in each test. Differential expression analysis for pseudo-cells was performed using generalized linear models as described previously^5^, with modifications to account for differential underlying count distributions in the ‘fit_models()’ function in Monocle3 (v.1.3.1)^2^.

### Spatial autocorrelation of transcriptional responses to perturbation

The local spatial statistic Getis–Ord index (*G*_i_)^66^ was used to identify statistically significant regions of the UMAP embedding that were enriched or depleted of perturbed cells. A high-value *G*_i_ indicates a perturbed cell is surrounded by other cells with the same perturbation, whereas a *G*_i_ close to zero indicates a perturbed cell is surrounded by cells with other perturbation labels. A *G*_i_ was calculated for each cell’s local neighbourhood (*k* = 15) using the ‘localG()’ function in the spdep package (v.1.2-8). This returns a *z* score that indicates whether the observed spatial clustering is more pronounced than expected by random. Multiple testing correction was performed using a Bonferroni correction. Areas of the UMAP where a given perturbation is enriched are called ‘hot spots’ while areas where a given perturbation is depleted are referred to as ‘cold spots’.

### Cell-count variance testing

We used above the beta-binomial generalized linear models (GLMs) for each cell type, to analyse their variability across individual embryos. At each timepoint, we calculated the coefficient of variation (coefficient of variation = *σ*/*μ*) for each cell type at each timepoint. We then regressed the cell type coefficient-of-variation values against their means with a gamma-valued GLM of the form identical to that of DESeq^11^ to capture the trend between the average number of cells in a cell type and that cell type’s coefficient of variation (with the VGAM package^67,68^, v.1.1-7). The curves in Fig. 1e illustrate the maximum likelihood estimate of a ‘typical’ cell type’s coefficient of variation at a given relative abundance, and the ribbon around it shows the 95% confidence interval of this estimate.

### Statistical assessment of cell-abundance changes

Changes in the proportions of each cell type were assessed by first counting the number of each annotated cell type in each embryo. To control for technical differences in cell recovery across embryos, ‘size factor’ normalization was performed by dividing the total number of cells recovered from an embryo by the geometric mean of total cell counts across all embryos. The number of cells of each type recovered from each embryo were then divided by that embryo’s size factor.

Normalized counts for each cell type *i* at time *t* were then compared across genotypes using a generalized linear model defined by the equations:

$${\rm{logit}}\left({\mu }_{i,t}\right)={\beta }_{t}+{\beta }_{g,t}{x}_{g}$$

$${\rm{logit}}\left({\rho }_{i,t}\right)={{\rm{\chi }}}_{t}+{{\rm{\chi }}}_{g,t}{x}_{g}$$

$${y}_{i,t}={\rm{BeBin}}\left({\mu }_{i,t},{\rho }_{i,t}\right)$$

Where *y*_*i,t*_, the normalized counts of cell type *i* at time *t* is modelled as a beta-binomially distributed random variable with mean *μ*_*i,t*_ and ‘litter effect’ *ρ*_*i,t*_ (that is, overdispersion with respect to the binomial distribution). We modelled both parameters of the beta-binomial response as a function of genotype, reasoning that crispants might exhibit greater variability than wild-type embryos. We also included the number of periderm cells as a nuisance term as a proxy for variation in overall animal size. The binary indicator variable *x*_*g*_ denotes whether gene *g* is knocked out in each embryo, and the corresponding *β*_*g*,*t*_ encodes the effect size on the relative abundance of the cell type at time* t*. Separate models for each gene in each cell type and at each timepoint were fit using the VGAM package (v.1.1-7)^69^. Significance of knockout effects in each model were assessed by Wald test on *β*_*g,t*_.

### Gene-set enrichment analyses

After differential expression testing, genes that had significant coefficients (*q* < 0.05) were used for gene-set enrichment analysis (GSEA) with the g:Profiler2 R package (v.0.2.1)^70^. Gene sets were filtered for significance (*q* < 0.01), and of the top gene sets, those having to do with neuronal development processes were chosen for visualization. For GSEA across all perturbations to look for generalized CRISPR–Cas9 editing effects, we averaged the normalized-effect scores across cell types and ranked the gene set by this averaged value for each perturbation. In this gene set, we included any gene that was called differentially expressed for at least one cell type and perturbation, which included over 10,000 ranked genes per perturbation. We performed GSEA using the msigdbr (https://davislaboratory.github.io/msigdb) and fgsea (v.1.26.0) R packages^71^ and the MSigDB ‘Hallmarks’ database via the msigdbR package (v.7.5.1)^72^, which summarizes 50 well-defined biological states and processes.

### Comparison of published zebrafish developmental atlases

Datasets for each study^7–9^ were downloaded. The authors of each dataset had used different naming conventions for gene names. To harmonize the datasets, the gene names from each dataset were first converted to the GRCz11 ENSEMBL gene names. Genes with duplicated names were removed and only genes found in all three datasets were retained. Datasets were then aligned with the IntegrateData function in Seurat V3. To compare wild-type transcriptomes at 24 hpf to stage-matched transcriptomes from refs. ^7–9^, wild-type reference data was first downsampled and then integrated using reciprocal PCA. Default hyperparameters were used for integration, PCA and dimensionality reduction. Following co-embedding, labels were transferred from refs. ^7–9^ to the wild-type reference data in the co-embedded space using the majority label from the 10 nearest neighbours. These labels were then used to calculate the concordance between the two datasets (Extended Data Fig. 1h).

### Statistics and reproducibility

For all WISH staining, the number of individuals analysed was at least ten.

### Reporting summary

Further information on research design is available in the Nature Portfolio Reporting Summary linked to this article.

## Online content

Any methods, additional references, Nature Portfolio reporting summaries, source data, extended data, supplementary information, acknowledgements, peer review information; details of author contributions and competing interests; and statements of data and code availability are available at 10.1038/s41586-023-06720-2.

### Supplementary information

Supplementary Protocol Protocol for zebrafish embryo sci-plex.

Reporting Summary

Supplementary Data Differentially expressed genes for each perturbation and cell type.

Supplementary Tables Supplementary Tables 1–8.

### Source data

Source Data Fig. 1

Source Data Fig. 2

Source Data Fig. 3

Source Data Fig. 4

Source Data Fig. 5

Source Data Extended Data Fig. 3

Source Data Extended Data Fig. 8

## Data Availability

The datasets generated and analysed during the current study are available in the NCBI Gene Expression Omnibus (GEO) repository under accession number GSE202639. The data have also been made available via their own website to facilitate their ongoing annotation by the research community at https://cole-trapnell-lab.github.io/zscape/. Source data not available via the GEO repository is available alongside the code at https://github.com/cole-trapnell-lab/sdg-zfish. The published datasets that were analysed for this study were accessed via either GEO repository GSE112294 or http://zebrafish-dev.cells.ucsc.edu^9^ and re-processed together. Published ISH images were downloaded from the ZFIN database^73^. Source data are provided with this paper.

## Code availability

Pipelines for generating count matrices from sci-RNA-seq3 sequencing data are available at https://github.com/bbi-lab/bbi-dmux and https://github.com/bbi-lab/bbi-sci. Analyses of the single-cell transcriptome data were performed using Monocle3; a general tutorial can be found at http://cole-trapnell-lab.github.io/monocle-release/monocle3. Analysis was performed in R and custom scripts can be found on GitHub at https://github.com/cole-trapnell-lab/sdg-zfish.
