# Supplementary material for: Embryo-scale reverse genetics at single-cell resolution
Source: Nature. 2023 Nov 15;623(7988):782–91. doi: 10.1038/s41586-023-06720-2 (PMC10665197; doi:10.1038/s41586-023-06720-2)
Supplement: Supplementary file 1 — Protocol for zebrafish embryo sci-plex. [file 41586_2023_6720_MOESM1_ESM.pdf]

nature portfolio

https://doi.org/10.1038/s41586-023-06720-2

Supplementary information

Embryo-scale reverse genetics at single-cell resolution

In the format provided by the authors and unedited

Protocol for sci-plex on individual zebrafish nuclei
Saunders, Srivatsan, *et. al.* (2022)

Materials

Reagents:

Nuclease free water (Thermo Fisher, cat. no. 10977015)

96-well V-bottom culture plates (Thermo Fisher, cat. no. 249952)

20% Paraformaldehyde Aqueous Solution, EM Grade (EMS, cat. no. 50-980-492)

100% Ethanol

Collagenase P (Millipore-Sigma, cat. no. 11213865001)

1X TrypLE Express (Thermo Fisher, cat. no. 12604013)

DPBS, no calcium, no magnesium (Thermo Fisher, cat. no. 14190144)

SUPERase In RNase Inhibitor (20 U/μL, Thermo Fisher, cat. no. AM2696)

BSA (20 mg/ml, NEB, cat. no. B9000S)

1M Tris-HCl (pH 7.5) (Thermo Fisher, cat. no. 15567027)

5M NaCl, RNase Free (Thermo Fisher)

1M $MgCl_2$, RNase Free (Thermo Fisher)

IGEPAL CA-630 (Millipore Sigma, cat. no. I8896)

Triton X-100 for molecular biology (Millipore Sigma, cat. no. T8787)

Superscript IV reverse transcriptase with 100mMDTT and buffer (Thermo Fisher, cat. no. 18090200)

10mM dNTP mix (NEB, cat. no. N0447L)

RNaseOUT Recombinant Ribonuclease Inhibitor (Thermo Fisher, cat. no. 10777019)

Quick Ligation Kit (NEB, cat. no. M2200L)

NEBNext High Fidelity 2x PCR master mix (NEB, cat. no. M0541L)

NEBNext mRNA Second Strand Synthesis Module (New England Biolabs, cat. no. E6111L)

Elution buffer (EB, 10mM Tris pH8.5, Qiagen, cat. no. 19086)

DNA Clean & Concentrator-5 (Zymo Research, cat. no. D4014)

Agencourt AMPure XP Beads (Beckman Coulter, cat. no. A63882)

DNA binding buffer (Zymo Research, cat. no. D4003-1-L)

Qubit dsDNA HS quantitation kit (Thermo Fisher, cat. no. Q32851)

N,N-Dimethylformamide (Millipore-Sigma, cat. no. D4551)

Liquid nitrogen for flash freezing

Tn5-N7 oligo (5′-GTCTCGTGGGCTCGGAGATGTGTATAAGAGACAG-3′, Eurofins, High-Purity Salt-Free)

Mosaic End (ME) oligo (5′-/5Phos/CTGTCTCTTATACACATCT-3′, Eurofins, High-Purity Salt-Free)

Plate(s) of indexed oligo-dT primers (5′-/5Phos/CAGAGCNNNNNNNN[10bpRTindex]TTTTTTTTTTTTTTTTTTTTTTTTTTTTTT-3′, where “N” is any base; IDT)

Plate(s) of indexed ligation primers (100uM, 5’- GCTCTG[9bp or 10bp barcode A]/ideoxyU/ACGACGCTCTTCCGATCT[reverse complement of barcode A]-3’)

Plates of Indexed PCR P5 primers (5′-AATGATACGGCGACCACCGAGATCTACAC[i5]ACACTCTTTCCCTACACGACGCTCTTCCGATCT-3′, IDT)

Plate of Indexed PCR P7 primers (5′-CAAGCAGAAGACGGCATACGAGAT[i7]GTCTCGTGGGCTCGG-3′, IDT)

Equipment:

DynaMag 96-Side Skirted Magnet (Thermo Fisher, cat. no. 12331D)

Refrigerated centrifuges that hold 1.5 ml microfuge tubes, microwell plates,15 ml and 50 ml conical tubes (i.e. Eppendorf, 5810R)

Low-bind tubes (Eppendorf, cat. no. 22431021)

Thermocycler that fits skirted 96-well plates (Bio-Rad, C1000)

Multichannel pipettes and tips

Hemocytometer

PCR plate seals (Thermo Fisher, catalogue #: AB0558)

96-well plates (Eppendorf, cat. no. 951020401)

96-well LoBind plates (Eppendorf, cat. no. 30129512)

Sonicator (Diagenode Bioruptor, model no. B01020001)

Thermomixer C + SmartBlock for microtiter plates (Eppendorf, cat. no. 5382000023 + cat. no. 5363000039)

Buffer Preparation

Collagenase P stock

Add 200 mg Collagenase P to 2 mL of nuclease-free water in a 15mL tube. Mix well until all chunks are resuspended – the suspension should be a dark brown. Make 100µL aliquots and store at -20ºC.

10% IGEPAL

5 mL IGEPAL CA-630 and 45mL nuclease-free water. Store at 4ºC.

10% Triton-X 100

5 mL Triton-X 100 and 45mL nuclease-free water. Store at 4ºC.

Nuclei Buffer (NSB)

10 mM Tris-HCl, pH 7.4, 10 mM NaCl, 3 mM $MgCl_2$.

To make 250 mL, mix 2.5 mL 1M Tris-HCl pH 7.5, 500 µL 5M NaCl, 750 µL 1M $MgCl_2$ and nuclease free water to 250 mL. Sterile filter and store at 4ºC.

Nuclei Suspension Buffer (NSB)

Make this buffer fresh each time. Choose the volume according to what you need. Nuclei buffer supplemented with 1% (v/v) SUPERase In and 1% (v/v) BSA. To make 5 mL, add 50µL SUPERaseIn and 50µL BSA to 5mL of nuclei buffer.

Hash Oligo plates

Add 10uL of NSB to the bottom of each well of a 96 well lo-bind plate. Add 5uL of 10uM hashing oligo to each well. These plates can be prepared in advance and stored at -20ºC prior to use

Cell Lysis Buffer (CLB)

Make this buffer fresh each time. Nuclei buffer with 0.1% (v/v) IGEPAL, 1% (v/v) SUPERaseIn RNase Inhibitor. Make enough CLB for the number of wells you plan to collect.

Per well, 35µL CLB = 0.5µl SUPERase In, 0.5µl 10% NP-40, and 34µl NSB (+ 15µl hash oligo in NSB).

To make barcoded lysis solution add 35µl CLB to 15µl in Hash Oligo plate (5µl 10µM hash, 10µl NSB, previously frozen).

Nuclei Buffer + BSA (NBB)

Mix 20 mL nuclei buffer and 200µL 20 mg/ml BSA (1% v/v). Store the buffer on ice.

Annealing N7 oligos

Tn5-N7	5′-GTCTCGTGGGCTCGGAGATGTGTATAAGAGACAG-3′
Mosaic End (ME)	5′-[phos]CTGTCTCTTATACACATCT-3′

Resuspend both oligos to 100µM in annealing buffer (50 mM NaCl, 40 mM Tris-HCl pH 8.0).

Mix one volume of Tn5-N7 with one volume of ME. This creates a working stock at 50 µM. Anneal them with the following PCR program: 95°C 5min, cool to 65°C (0.1°C/sec), 65°C 5min, cool to 4°C (0.1°C/sec). Store annealed oligos at 4°C or aliquot and freeze at -20°C.

N7-loaded Tn5

Tagmentase (Tn5 transposase) - unloaded (Diagenode cat. no. C01070010-20).

To 20µl of Tn5, add 20µl of annealed N7 oligos. Place in a thermomixer and shake at 350 rpm, 23°C, 30 minutes.

Add 20µl of glycerol. Store at -20°C.

Tagment DNA (TD) buffer (2X)

For a final volume of 50mL, add 1 mL 1M Tris pH 7.6, 250 µL of 2M $MgCl_2$, 10 mL dimethylformamide, to 38.75 ml of nuclease-free water.

Make 550 µL aliquots and store at -20°C.

Indexed Primer Plates

Primers for reverse transcription, ligation, and PCR indexing steps are at 100 µM. Store at 4°C, make sure plates are well sealed between uses.

Protocol

General Notes

Everything needs to be kept cold at all times. In preparation for an experiment, pre-chill centrifuges and get plenty of ice ready. Pre-cool all tubes on ice before adding nuclei to them.

Prepare a clean workspace before each experiment. Use RNAseZap to clean benches, pipettes, racks and centrifuges.

Use low-bind plastics for all steps involving nuclei.

Nuclei isolation and fixation from individuals

Timing: 2 hours

1. In a 96-well V-bottom plate, to each well, add 1 embryo, 73.5 µL of 1X TrypLE and 1.5µL Collagenase P (100 mg/mL). Note: First prep the V-bottom plate by adding 43.5µL of TrypLE and 1.5µL Collagenase to each well on ice. Then move embryos into a petri dish containing TrypLE to dilute out embryo media. Then move each embryo along with 30uL of TrypLE with a wide-cut pipette tip to each well of a 96-well V-bottom plate.
2. Incubate at 30°C and pipette the tissue every 3-5 minutes to produce a single cell suspension. Note: incubation time varies depending on embryo age. A 24 hpf embryo takes ~20 minutes, while a 96 hpf embryo may takes closer to ~35 minutes. When samples are fully dissociated, there should be no visible tissue pieces under a dissecting scope. Bubble should be minimized during the dissociation process, but a few are ok.
3. After embryos are fully dissociated, add 180 µL of cold 10% FBS (v/v) in dPBS to each well. Pellet the cells (600g, 5min) and remove the supernatant carefully with a multichannel pipette.
4. Add 180µL of cold dPBS to each well. Pellet cells again to rinse (600g, 5min) and remove ALL supernatant carefully with a multichannel pipette.
5. Before continuing to step 6, make sure hash oligo plate and 5% PFA solutions have been prepared in advance (see buffer preparation section).
6. Add 50µL of diluted hashes in CLB to each well and pipette to mix (~3 times). Incubate on ice for 3 min. Return tips to box to save for mixing fixative. Tips are saved to reduce tip waste – each tip should only see 1 well.

7. Add 200µL of ice cold 5% PFA solution to each well. Mix by pipetting 2-3 times using hashing tips making sure not to switch tip-well locations! Fix the nuclei on ice for 15 minutes (do not replace lid of 96 well V-bottom plate during fixation).
8. Pool fixed nuclei in 15 mL conical tube(s). Pellet the nuclei (750g, 15 min) and remove the supernatant by decanting.
9. Re-suspend the nuclei with 2 mL NSB and mix briefly. Pellet the nuclei (750g, 6min) and remove supernatant with a pipette.
10. Re-suspend the nuclei with 500µL NSB. Flash freeze the nuclei (still in 15 mL conical tubes) in liquid nitrogen and store at -80°C. Nuclei can stay frozen for weeks to months.

Stopping point.

Nuclei thawing and permeabilization

Timing: 1 hour

11. Thaw fixed nuclei on ice with intermittent mixing and warming by hand. Pool samples if thawing multiple; you may want to make two pools for balancing the centrifuge – you can combine them later. Before combining samples note the below considerations:
    a. If the cells were barcoded with different hash oligos they can be combined at this stage.
    b. If any two conditions received the same hash oligos, those samples should not be combined at this stage, they should be kept separate and loaded into different RT wells.
12. Pellet the nuclei (750g, 6 min, 4°C) and remove the supernatant. You should see a pellet – be careful not to disturb it.
13. For each sample tube, resuspend the nuclei in 100µL NSB. For each sample, then add a mix containing 390µL NSB and 10µL 10% Triton X-100. This step is crucial for permeabilizing nuclei.
14. Incubate the mix on ice for 3 min.
15. Pellet the nuclei (750g, 6 min), and remove the supernatant. Resuspend the nuclei in 400µL NSB.
16. Perform sonication in Bioruptor Sonication device (low power, 12 sec). We have seen that sonication increases the number of single nuclei recovered from tissue, while having a minimal effect on the number of UMIs recovered per cell.
17. Pellet the nuclei (750g, 6 min), and remove the supernatant.
18. Resuspend the nuclei in 100-500µL NSB and count nuclei with a hemacytometer. Pool samples here if they were kept separate at step 11.

Reverse transcription (RT)

Timing: 2-3 hours

19. Follow the chart below to determine how many starting nuclei that you need and their volume. In each well: 25,000 nuclei in 22µL NSB and 2µL dNTPs. The below are calculated with ~10% extra volume for pipetting error.

**Cell number:**	**2M**	**1M**	**800K**	**400K**	**200K**
**Number of columns:**	12	6	4	2	1
**Nuclei volume:**	2240µL	1166µL	770µL	396µL	176µL
**10mM dNTPs:**	220µL	106µL	70µL	36µL	16µL

20. Resuspend the nuclei in the necessary volume according to the chart and add the corresponding amount of dNTPs.
21. Dispense 24µL of the nuclei/dNTP mix into each well of a low-bind 96-well plate, on ice.
22. Spin the plate of 3-level RT primers (100uM) briefly to bring down any condensate on seal. Be careful when removing seal not to cross contaminate any of the oligos while opening.
23. Add 2µL of primer to each well and stir gently to mix (do not pipette up and down).
24. Incubate the plates at 55°C (heated lid at 65°C) for 5 min. Immediately place the plates on ice.
25. While the plates are incubating, prepare the reaction mix:

RT mix:	1 well	x110 (1 plate)	x410 (4 plates)
5X Superscript IV buffer	8µL	880µL	3,280µL
100 mM DTT	2µL	220µL	820µL
SuperScript IV (200 u/µL)	2µL	220µL	820µL
RNaseOUT	2µL	220µL	820µL

26. Mix the RT master mix well and distribute 14µL to each well. Don't forget to mix!

27. Incubate the RT reaction with the following program:

    4°C 2 minutes
    10°C 2 minutes
    20°C 2 minutes
    30°C 2 minutes
    40°C 2 minutes
    50°C 2 minutes
    55°C 15 minutes
    Hold at 10°C

28. After the reaction, add 60µL NBB into each well. Pool the nuclei from all wells and distribute into two 15 mL conical tubes.

29. Pellet the nuclei (750g, 10min), and remove supernatant.

Ligation

Timing: 1 hour

30. Resuspend the cells in 4.3mL NSB and distribute 10µL into each well of 4 x 96-well plates.

31. Quick spin the plate(s) of ligation primers (100uM).

32. Add 8µL indexed ligation primer into each well.

33. Prepare ligation master mix. **Mix well**.

Ligation mix:	1 well	x110 (1 plate)	x410 (4 plates)
2X Quick ligase buffer	20µL	2,200µL	8,200µL
Quick ligase enzyme	2µL	220µL	820µL

34. Distribute 22µL of the ligation master mix into each well.

35. Incubate the ligation at 25°C for 10 min.

36. After the reaction, add 60µL NBB into each well. Pool the nuclei from all wells and distribute into a single 50mL conical tube.

37. Add another 40mL NBB to the nuclei mix. Pellet the nuclei (800g, 10 min) and decant the supernatant.

38. Resuspend the cells in 5mL NBB. Pellet the nuclei (800g, 10 min) and decant the supernatant.

39. Resuspend cells in 500µL NBB.

40. Count the nuclei concentration with hemacytometer. You may need to pass the nuclei through a 40uM cell strainer if there are still lots of clumps.

41. Resuspend the nuclei in the proper volume to distribute 2500 to 4000 nuclei per well in 5µL total volume/well.

42. The plates can be frozen in -80°C for at up to 1 month.

Stopping point.

Second strand synthesis

Timing: 3 hours

43. If plates were frozen, thaw one plate (with nuclei distributed per well) at room temperature and directly plate on ice. If proceeding directly, perform second strand synthesis on one plate as a test before prepping the others (freeze them).

44. Prepare second strand synthesis reaction mix:

Second strand mix:	1 well	x110 (1 plate)
Elution buffer	3µL	330µL
10X Second strand buffer	1.33µL	146.7µL
Second strand enzyme	0.75µL	73.3µL

45. Distribute 5µL reaction mix per well. Mix and briefly centrifuge the plate.

46. Perform second strand synthesis at 16°C for 3 hours.

Tagmentation

Timing: 15 min

47. Prepare the tagmentation mix by combining 1.1 mL tagmentation buffer, 1µL N7 adaptor loaded Tn5 and mix well. It is best to do a dilution series of Tn5 in a single column of post-second strand nuclei (separate strip tube pipetted out at same time as plate(s)) to select the proper Tn5 concentration for the rest of the plate.
    a. A well tagmented library:

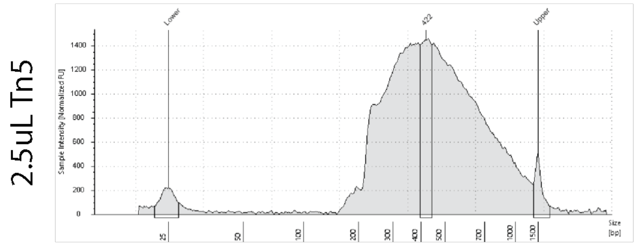

An under-tagmented library:

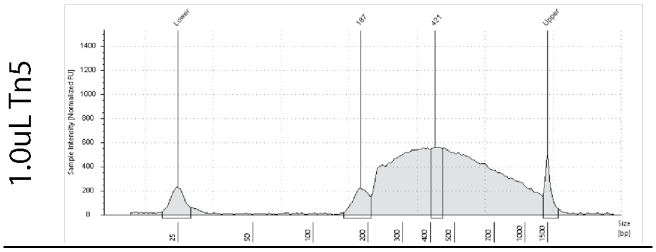

Tn5 concentrations tested on single wells. Traces are captured on the tape station and derive from the perturbation sequencing libraries produced in this paper. Amount of Tn5 indicated corresponds to the amount that would have been used for a whole plate.

48. Briefly centrifuge the plate and add 10µL reaction mix into each well.
49. Perform tagmentation at 55°C at 5min.
50. Add 20µL of Zymo DNA binding buffer per well, and incubate the reaction mix at room temperature for 5 min.

Ampure bead purification

Timing: 30 min

51. Prepare fresh 80% ethanol. You will need ~22mL per plate.
52. To each well, add 60µL of Ampure XP beads (1.5X SPRI) and mix well.
53. Incubate the mix at room temperature for 5 min.

54. Put the plate on magnetic stand for 5min, until the supernatant is clear and remove supernatant.
55. Wash each well with 100µL 80% ethanol.
56. Remove ethanol, and wash again with 100µL of 80% ethanol.
57. After the second wash, carefully remove all supernatant from each well and dry the plate at room temperature for 3 min.
58. Add 18µL elution buffer to each well, mix well and allow 2 min for elution.
59. Briefly centrifuge the plate and leave the plate on magnetic stand for 3 min.
    a. This eluate will be transferred to a new 96-well plate containing PCR primers and master mix (Step 56).
    b. Until the PCR plate has been set up (steps 56 and 57), allow the plate to remain on the magnet

PCR amplification

Timing: 2 hours

60. To each well of a fresh 96-well plate (low-bind not necessary), add 2µL P5 PCR primer (10uM) directly to the bottom of the well.
61. To each well of a 12-well strip tube (8 + 4 wells), add 170µL NEBnext Master Mix and 17µL P7 PCR primer (10µM, 12 separate oligos from 1 row of PCR primer plate). Using a 12-well multichannel pipette, add 22µL of this mix to the front wall of each well without changing pipette tips.
62. Transfer 16µL of the eluate from step 54 to each well of the PCR reaction plate and mix well by pipetting.
63. Amplify the libraries with the following program:

Step 1 - 72°C for 5 min
Step 2 - 98°C for 30 sec
Step 3 - 98°C for 10 sec
Step 4 - 66°C for 30 sec
Step 5 - 72°C for 30 sec
Return to step 3 for 12 to 15 cycles
Step 6 - 72°C for 5 min.

Stopping point.

    c. Libraries are stable and can be stored for up to a week at 4°C or at -20°C for longer stretches of time.

64. Pool all wells together (the final volume for 1 plate will be ~3.8mL), and take 1mL PCR product for a 1X Ampure XP bead purification. Elute the product in 50µL elution buffer.
65. Purify the library with a Zymo clean-up column purification.
66. Determine library concentration with Qubit dsDNA HS.
67. Estimate library size using an Agilent Tapestation or Bioanlyzer. The mean fragment size should be around 450bp.
68. Sequence the library on a NextSeq 2000 (or NovaSeq 6000) using standard Illumina sequencing primers and a 100-cycle kit.

    Read1: 34 cycles
    Index1: 10 cycles
    Index2: 10 cycles
    Read2: 84 cycles

    If sequencing on a NextSeq500/550, use a 150-cycle kit with the following chemistry.

    Read1: 34 cycles
    Index1: 10 cycles
    Index2: 10 cycles
    Read2: 112 cycles
