# Supplementary material for: Embryo-scale reverse genetics at single-cell resolution
Source: Nature. 2023 Nov 15;623(7988):782–91. doi: 10.1038/s41586-023-06720-2 (PMC10665197; doi:10.1038/s41586-023-06720-2)
Supplement: Supplementary file 2 — Reporting Summary [file 41586_2023_6720_MOESM2_ESM.pdf]

nature portfolio

Corresponding author(s): Cole Trapnell, David Kimelman

Last updated by author(s): Sep 24, 2023

Reporting Summary

Nature Portfolio wishes to improve the reproducibility of the work that we publish. This form provides structure for consistency and transparency in reporting. For further information on Nature Portfolio policies, see our Editorial Policies and the Editorial Policy Checklist.

Statistics

For all statistical analyses, confirm that the following items are present in the figure legend, table legend, main text, or Methods section.

n/a	Confirmed	
☐	☒	The exact sample size (*n*) for each experimental group/condition, given as a discrete number and unit of measurement
☐	☒	A statement on whether measurements were taken from distinct samples or whether the same sample was measured repeatedly
☐	☒	The statistical test(s) used AND whether they are one- or two-sided<br>*Only common tests should be described solely by name; describe more complex techniques in the Methods section.*
☐	☒	A description of all covariates tested
☐	☒	A description of any assumptions or corrections, such as tests of normality and adjustment for multiple comparisons
☐	☒	A full description of the statistical parameters including central tendency (e.g. means) or other basic estimates (e.g. regression coefficient) AND variation (e.g. standard deviation) or associated estimates of uncertainty (e.g. confidence intervals)
☐	☒	For null hypothesis testing, the test statistic (e.g. *F*, *t*, *r*) with confidence intervals, effect sizes, degrees of freedom and *P* value noted<br>*Give P values as exact values whenever suitable.*
☒	☐	For Bayesian analysis, information on the choice of priors and Markov chain Monte Carlo settings
☐	☒	For hierarchical and complex designs, identification of the appropriate level for tests and full reporting of outcomes
☐	☒	Estimates of effect sizes (e.g. Cohen's *d*, Pearson's *r*), indicating how they were calculated

Our web collection on statistics for biologists contains articles on many of the points above.

Software and code

Policy information about availability of computer code

Data collection	No software was used for data collection in this study.
Data analysis	For single cell RNA-seq data processing and count matrix generation, we used open source pipelines (https://github.com/bbi-lab/bbi-dmux; https://github.com/bbi-lab/bbi-sci). Analyses of the single cell transcriptome data were performed using Monocle3; a general tutorial can be found at http://cole-trapnell-lab.github.io/monocle-release/monocle3. Analysis was performed in R, and custom scripts can be found on Github at https://github.com/cole-trapnell-lab/sdg-zfish. The following R package versions were used for analyses: monocle3 v1.3.1, VGAM v1.1-7, spdep v1.2-8, RcppAnnoy v0.0.20, gProfiler2 v0.2.1, amplican v1.22.1, fgsea v1.26.0, msigdbR v7.5.1.

For manuscripts utilizing custom algorithms or software that are central to the research but not yet described in published literature, software must be made available to editors and reviewers. We strongly encourage code deposition in a community repository (e.g. GitHub). See the Nature Portfolio guidelines for submitting code & software for further information.

Data

Policy information about availability of data

All manuscripts must include a data availability statement. This statement should provide the following information, where applicable:

- Accession codes, unique identifiers, or web links for publicly available datasets
- A description of any restrictions on data availability
- For clinical datasets or third party data, please ensure that the statement adheres to our policy

The datasets generated and analyzed during the current study are available in the NCBI Gene Expression Omnibus (GEO) repository under accession number GSE202639. The data have also been made available via their own website to facilitate their ongoing annotation by the research community at https://cole-trapnell-

lab.github.io/zscape/. The published datasets that were analyzed for this study were accessed via either GEO repository GSE112294 or http://zebrafish-dev.cells.ucsc.edu, and reprocessed together. Published in situ hybridization images were downloaded from the ZFIN database.

Field-specific reporting

Please select the one below that is the best fit for your research. If you are not sure, read the appropriate sections before making your selection.

☒ Life sciences ☐ Behavioural & social sciences ☐ Ecological, evolutionary & environmental sciences

For a reference copy of the document with all sections, see nature.com/documents/nr-reporting-summary-flat.pdf

Life sciences study design

All studies must disclose on these points even when the disclosure is negative.

Sample size	Sample sizes of individual embryos sampled for single cell RNA-seq were chosen based on pilot experiments in which we calculated our ability to detect statistically significant changes in the abundances of cell types across a range of mean abundances and effect sizes via a beta binomial regression model. We sampled an average of 8 individuals per condition based on these calculations, because they predicted that we could identify 25% effect size changes in rare cell populations given a empirically determined distribution. Sample sizes for imaging-based cell count studies were targeted at the same number, and statistics were performed only after all images were analyzed.
Data exclusions	Excluded data are cells that did not pass filtering metrics for single cell RNA-seq analysis. We established these cutoffs empirically for low and high UMI counts as well as mitochondrial read fraction. Excluded cells were filtered out prior to all published analyses and conclusions.
Replication	We included between 8-48 biological replicates for each embryo collected in the single cell experiments. For other experiments, such as analyzing gene expression via ISH, we included at least 10 replicate embryos and took all of the data into account when making conclusions and selecting representative images. All attempts at replication were successful.
Randomization	For each scRNA-seq experiment outlined in the manuscript, groups of embryos were dissociated in parallel, nuclei fixed and then all samples were pooled (i.e. randomly combined). This approach offers a substantial advantage over most droplet based scRNA-seq approaches, as all individually labeled embryos are exposed to the same library preparation procedure. Because of sci-plex hashing, sample labels can be resolved computationally after sequencing. All directly-compared samples were from the same single cell RNA-sequencing experiment, which reduces the effect of technical batch effects (i.e. overall cell recovery per embryo) for statistics measuring differences in cell abundance or perturbation-dependent gene expression differences. We looked for possible batch effects across experiments in our wildtype atlass (note that samples have some timepoint overlap but are not pure replicates), and we did not see experiment-specific effects on transcriptomes. These results are described in the Extended data.
Blinding	Blinding was done for cell counts and area measurements of cranial ganglia confocal images. No other measurements were done manually.

Reporting for specific materials, systems and methods

We require information from authors about some types of materials, experimental systems and methods used in many studies. Here, indicate whether each material, system or method listed is relevant to your study. If you are not sure if a list item applies to your research, read the appropriate section before selecting a response.

Materials & experimental systems

n/a	Involved in the study	
☐	☒	Antibodies
☒	☐	Eukaryotic cell lines
☒	☐	Palaeontology and archaeology
☐	☒	Animals and other organisms
☒	☐	Human research participants
☒	☐	Clinical data
☒	☐	Dual use research of concern

Methods

n/a	Involved in the study	
☒	☐	ChIP-seq
☒	☐	Flow cytometry
☒	☐	MRI-based neuroimaging

Antibodies

Antibodies used	a mouse mAb anti-HuC/D (aka. elavl) primary antibody (16A11, Thermo Fisher, 1:750) with Goat anti-Mouse IgG Alexa Fluor 647 (Thermo Fisher, A21236, 1:400).
Validation	This antibody has been used extensively by our co-authors and is used in multiple publications in zebrafish, including PMID: 16364284, PMID: 28708822, PMID: 22738203.

Animals and other organisms

Policy information about studies involving animals; ARRIVE guidelines recommended for reporting animal research

Laboratory animals	Danio rerio; strains used were were: wild-type AB, noto-n1, tbx16-b104, Tg(isl1:gfp)-rw, Tg(p2rx3:gfp)-sl1, mafba-b337, hgfa-fh528, met-fh533; ages ranged from 12 to 96 hours post fertilization. For imaging analyses, Tg(sox10:nlsEos)-w18 was used, and animals were sacrificed at 72 hpf.
Wild animals	No wild animals were used in this study.
Field-collected samples	No field-collected samples were used in this study.
Ethics oversight	All procedures involving live animals followed federal, state and local guidelines for humane treatment and protocols approved by Institutional Animal Care and Use Committees of the University of Washington and the Fred Hutchinson Cancer Research Center.

Note that full information on the approval of the study protocol must also be provided in the manuscript.
